# Deregulation of the EGFR/PI3K/PTEN/Akt/mTORC1 pathway in breast cancer: possibilities for therapeutic intervention

**DOI:** 10.18632/oncotarget.2209

**Published:** 2014-07-12

**Authors:** Nicole M. Davis, Melissa Sokolosky, Kristin Stadelman, Stephen L. Abrams, Massimo Libra, Saverio Candido, Ferdinando Nicoletti, Jerry Polesel, Roberta Maestro, Antonino D’Assoro, Lyudmyla Drobot, Dariusz Rakus, Agnieszka Gizak, Piotr Laidler, Joanna Dulińska-Litewka, Joerg Basecke, Sanja Mijatovic, Danijela Maksimovic-Ivanic, Giuseppe Montalto, Melchiorre Cervello, Timothy L. Fitzgerald, Zoya N. Demidenko, Alberto M. Martelli, Lucio Cocco, Linda S. Steelman, James A. McCubrey

**Affiliations:** ^1^ Department of Microbiology and Immunology, Brody School of Medicine at East Carolina University Greenville, NC 27858 USA; ^2^ Department of Bio-Medical Sciences, University of Catania, Catania, Italy; ^3^ Unit of Epidemiology and Biostatistics, Centro di Riferimento Oncologico, IRCCS, Aviano, Italy; ^4^ Experimental Oncology 1, CRO IRCCS, National Cancer Institute, Aviano, Pordenone, Italy; ^5^ Department of Biochemistry and Molecular Biology, Mayo Clinic College of Medicine, Rochester, MN, USA; ^6^ Palladin Institute of Biochemistry, National Academy of Sciences of Ukraine, Kyiv, Ukraine; ^7^ Department of Animal Molecular Physiology, Institute of Experimental Biology, Wroclaw University, Wroclaw, Poland; ^8^ Chair of Medical Biochemistry, Jagiellonian University Medical College, Kraków, Poland; ^9^ Department of Medicine University of Göttingen, Göttingen, Germany; ^10^ Department of Immunology, Institute for Biological Research “Sinisa Stankovic” University of Belgrade, Belgrade, Serbia; ^11^ Biomedical Department of Internal Medicine and Specialties, University of Palermo, Palermo, Italy; ^12^ Consiglio Nazionale delle Ricerche,Istituto di Biomedicina e Immunologia Molecolare “Alberto Monroy”, Palermo, Italy; ^13^ Department of Surgery, Brody School of Medicine at East Carolina University, Greenville, NC; ^14^ Department of Cell Stress Biology, Roswell Park Cancer Institute, Buffalo, NY, USA; ^15^ Dipartimento di Scienze Biomediche e Neuromotorie, Università di Bologna, Bologna, Italy

**Keywords:** Targeted Therapy, Therapy Resistance, Mutations, PI3K, mTOR, rapamycin

## Abstract

The EGFR/PI3K/PTEN/Akt/mTORC1/GSK-3 pathway plays prominent roles in malignant transformation, prevention of apoptosis, drug resistance and metastasis. The expression of this pathway is frequently altered in breast cancer due to mutations at or aberrant expression of: *HER2*, *ER*alpha, *BRCA1, BRCA2, EGFR1*, *PIK3CA*, *PTEN*, *TP53*, *RB* as well as other oncogenes and tumor suppressor genes. In some breast cancer cases, mutations at certain components of this pathway (*e.g.*, *PIK3CA*) are associated with a better prognosis than breast cancers lacking these mutations. The expression of this pathway and upstream HER2 has been associated with breast cancer initiating cells (CICs) and in some cases resistance to treatment. The anti-diabetes drug metformin can suppress the growth of breast CICs and herceptin-resistant HER2+ cells. This review will discuss the importance of the EGFR/PI3K/PTEN/Akt/mTORC1/GSK-3 pathway primarily in breast cancer but will also include relevant examples from other cancer types. The targeting of this pathway will be discussed as well as clinical trials with novel small molecule inhibitors. The targeting of the hormone receptor, HER2 and EGFR1 in breast cancer will be reviewed in association with suppression of the EGFR/PI3K/PTEN/Akt/mTORC1/GSK-3 pathway.

## INTRODUCTION

Breast cancer is a leading cause of cancer-related death in women. This disease is diagnosed in nearly 1.4 million women and is responsible for more than 450,000 death every year [1]. According to the WHO release, there has been a 20% increase in the number of reported worldwide breast cancer patients which resulted in 522,000 deaths since 2008. According to the US National Cancer Institute, approximately 232,000 occur resulting in about 40,000 deaths in the USA each year. Breast cancer is not gender specific [2,3]. The frequency of breast cancer in men is approximately 100-fold lower than in women as approximately 2,200 males will be diagnosed with breast cancer each year in the USA.

### Breast Cancer Genetics

A prominent risk factor for the onset of breast cancer is age; however factors linked to lifestyle and diet also play important roles in breast cancer. Mutations at or deregulation of certain genes (*BRCA1, BRCA2, HER2, PIK3CA*) and others play important roles in breast cancer [4-18]. Mutations or aberrant or deregulated expression of *TP53*, *MDM2* and *RB* also can play roles in the therapeutic responses of breast cancer [19-26]. Restoration of functional TP53 activity can increase the sensitivity of some *TP53* mutant cells to certain anticancer drugs [27].

### BRCA Genes and Other Genes Involved in DNA Repair Are Implicated in Breast Cancer

Breast cancer occurrence is attributed to both genetic and environmental factors. Some breast cancers are due to hereditary mutations, namely those involving *BRCA1* and *BRCA2. BRCA1* encodes breast cancer type 1 susceptibility protein which is involved in DNA repair and is considered a caretaker gene. The BRCA1 protein interacts with RNA polymerase II and also with histone deacetylase complexes [28]. BRCA1 plays key roles in transcription, repair of breaks in double stranded DNA as well as ubiquitination. The BRCA1 protein also combines with other proteins which detect DNA damage and other cell signals and forms a multi-subunit protein complex known as the BRCA1-associated genome surveillance complex (BASC) [29]. Components of this complex may be mutated in certain cancers.

BRCA2 is also involved in the repair of DNA double strand breaks [30]. BRCA2 binds single stranded DNA. BRAC2 interacts with the RAD51 recombinase to stimulate strand invasion which is a critical step in homologous recombination. For RAD51 to bind the DNA double-strand breaks, a complex of BRCA1/partner and localizer of BRCA2 (PALB2)/BRCA2 is required [31].

The risk of developing breast or ovarian cancer in individuals with certain cancer-associated *BRCA1/BRCA2* alleles is 60-80% for breast cancer and 20-40% for ovarian cancer. These individuals also develop cancer at an earlier age. In addition, other genes involved in DNA repair and signaling are implicated in breast cancer including: Fanconi anemia (FA) genes (*FANCD2*, *FANCA* and *FANCC*), mismatch repair genes (MutL homolog 1 [*MLH1*], MutS protein homolog 2 [*MSH2*], PMS1 protein homolog 1 [*PMS1*], mutS homolog 6 [*MSH6*]), mismatch repair endonuclease PMS2 [*PMS2*] and DNA repair genes (Ataxia telangiectasia mutated [*ATM*], Ataxia telangiectasia and Rad3 related [*ATR*] and serine/threonine-protein kinases Chk/2 (*CHK1/2*), and the tumor suppressor genes (*TP53*, Serine/threonine kinase 11 [*STK11*] also known as liver kinase B1[*LKB1*], phosphatase and tensin homolog [*PTEN*]) and protein phosphatase 6 (*PP6*) [32-44].

In an important study with triple negative breast cancer (TNBC) patients, the frequency of *BRCA1* and *BRCA2* mutations and survival was examined [45]. DNA was isolated from tumor samples as well as normal tissues from 77 TNBC patients and the genetic sequence of the *BRCA1/2* exons and flanking regions determined. 19.5% of the TNBC patients had *BRCA* mutations, 15.6% were mutant at *BRCA1*, and 3.9% were mutant at *BRCA2*. It turns out that the patients with *BRCA* mutations were younger than the patients with WT *BRCA* genes. In this study which followed the patients for up to 214 months, there were 42.9% recurrences and 45.5% deaths. Interestingly, the five-year recurrence-free survival estimates were associated with the genetic status of the *BRCA* genes. As the five-year recurrence-free survival rates were 51.7% for patients with WT *BRCA* genes whereas they were 86.2% for patients with *BRCA* mutations.

*BRCA1* and *BRCA2* are also mutated in patients with ovarian cancer [46]. *BRCA1/2* mutations are present in approximately 11 to 15% of unselected ovarian cancer patients. *BRCA1* mutations were positively associated with *TP53* mutations. The presence of *BRCA1/2* mutations after platinum chemotherapy were associated with improved progression free survival.

### Hereditary and Sporadic Breast and Ovarian Cancer

Many spontaneous breast cancers are associated with environmental exposures to carcinogens [47-61]. These include: air pollution [52], exposure to polychlorinated biphenyl congeners [53]. Pesticides [54,58], electromagnetic radiation [55], cadmium and nickel [56], radiation from medical imaging [59], acrylamide [61] and other toxins.

Deregulation of BRCA1 expression has been implicated in sporadic breast cancer. The trinucleotide-repeat-containing 9 (*TNRC9*) gene has been shown to downregulate BRCA1 expression which results in breast cancer aggressiveness. *TNRC9* is amplified in certain breast cancer patients and is associated with a poor prognosis [62]. This group also determined that ectopic expression of TNRC9 affected breast cancer cell survival. TNRC9 and BRCA1 protein expression were inversely correlated in large data sets of breast and ovarian cancer samples. Interesting this group determined that TNRC9 bound to both the *BRCA1* promoter and the cAMP-responsive element-binding protein (CREB) complex. CREB is a regulator of BRCA1 transcription. Finally TNRC9 expression suppressed BRCA1 expression by altering the methylation status of the *BRCA1* promoter region.

*BRCA* mutations have also been detected in familial and sporadic ovarian cancer patients. Germline mutations in *BRCA1* or *BRCA2* are present in approximately 18% of hereditary ovarian cancers. These mutations confer an estimated risk from 15 to 50% in the ovarian cancer patients [63].

In this study, the prevalence of *BRCA1* mutations in 106 familial Greek ovarian cancer patients who had a strong family history of ovarian cancer or metachronous breast cancer. Metachronous breast cancer refers to a breast cancer patient which has two different breast cancers which occur at two different times, the two cancers can occur in the same breast. In addition, the prevalence of *BRCA1* mutations were examined in 592 sporadic Greek ovarian cancer patients. In Greece, it had been previously determined that there were 6 types of *BRCA1* mutations that accounted for 63% of all the mutations in the *BRCA1* and *BRCA2* genes. Deleterious *BRCA1* mutations were observed in 40.6% of familial ovarian cancer cases and 4.6% of sporadic ovarian cases. This study determined that 71.2% of the *BRCA1* carriers presented a high-grade serous phenotype. These studies document the importance of identifying *BRCA* mutations in breast and/or ovarian cancer families. The authors have stated that all serous ovarian cancer patients should consider genetic testing.

Hereditary breast cancer often results from disruption of the normal functions of *BRCA1* and *BRCA2*. In contrast *BRCA1/BRCA2* are not necessarily mutated in sporadic breast cancer, but there may be mutations in *TP53* and epigenetic alterations which change the expression of other genes. These changes result in breast cancer cells which may be wild WT at *BRCA1* and *BRCA2* but have defects in DNA repair. In addition certain sporadic breast cancers may have hypermethylation of the *BRCA1*, estrogen receptor (*ER*), progesterone receptor (*PR*) and other genes which prevents or lowers their expression. These changes may results in the breast cancer cells having a mutant *BRCA* phenotype which is referred to as “BRCAness”. These breast cancers arise at early age and are aggressive [64].

In one study, the frequencies of methylation in the *BRCA1* promoter region were examined in 96 sporadic invasive breast carcinomas and 43 sporadic ovarian carcinomas. Methylation of the *BRCA* promoter region was detected in 11% and 5% of the breast and ovarian carcinomas respectively. Methylation of the *BRCA1* promoter was linked with lack of ER and PR expression in these tumors [65].

### Alterations of Genes Involved in DNA Repair in Breast Cancer

Sporadic breast cancers accounts for approximately 70% of breast cancers, while familiar breast cancers account for the remaining 30% which are due to the present of mutations in breast cancer families (familiar breast cancers) [66]. Familial breast cancer families have a higher incidence of breast cancers. There exist different susceptibility genes which are high-, moderate-, and low-penetrance susceptibility genes. High-penetrance genes include: *BRCA1*, *BRCA2*, *PTEN* and *TP53*, which in many cases are responsible at least in part for the familiar breast cancer. Other genes have been associated with moderate penetrance and risk. These include genes involved in DNA repair, such as *CHEK2*, *ATM*, *BRIP1* (*FANCJ*), *PALB2* (*FANCN*) and *RAD51C* (*FANCO*). In addition, genes involved in low penetrance and risk are being identified. The presence of mutations at these different types of breast cancer susceptibility genes may be examined in breast cancer screening in the future.

The genetic structures of the *TP53*, *BRCA1*, *ATM*, and *PIK3CA* genes were examined in 145 Bulgarian patients with sporadic breast cancer. The expression of *HER2* was examined by immunohistochemistry and chromogenic hybridization *in situ* (CISH) [67]. In this study, mutations were observed: at *TP53* in 22.07% of the patients, at *BRCA1* in 0.69% of the patients, at *ATM* in 7.59% of the patients, and in 31.25% of the patients at *PIK3CA*. Overexpression of HER2 was observed in 21.21% of the patients. Mutations at *TP53* were associated with both tumor size and grade of malignancy. Mutations at *ATM* were associated with grade of malignancy. Mutations at *PIK3CA* were associated with PR+ tumors. HER2 overexpression was correlated with the age of the patient when diagnosed with breast cancer, tumor size and ER+. This important study documented that *TP53* mutations were an indicator for poorer outcome. Importantly, in this study the presence of two genetic mutations did not correlate with either a more aggressive carcinoma or a poorer overall survival.

Chk2 is an important kinase which is activated in response to DNA damage. Chk2 is involved both in TP53 and cell-cycle pathways. In some Li-Fraumeni syndrome families, which have germline mutations at *TP53*, mutations at *CHK2* have also be observed. The presence of loss of heterozygosity (LOH) at the *CHK2* gene was examined in 139 breast cancer tumors [68]. 139 breast tumors were screened for loss of heterozygosity (LOH) at chromosome 22q (where the *CHK2* gene is located), using seven microsatellite markers, LOH was detected in 53% of the breast tumors. Further studies examined the mutational status of the *CHK2* gene and a germ line variant (T59K) in the first coding exon was detected. Upon screening 1172 cancer patients with different types of cancer for the T59K sequence variant, it was detected in four breast cancer patients. This study concluded that *CHK2* mutations were rare in breast cancer but the *CHK2* gene product may perform a tumor suppressor function.

### Effects of c-Myc Overexpression in BRCA1-Deficient Breast Cancer Patients

Elevated c-Myc expression leads to a poor prognosis in sporadic breast cancer patients that are *BRCA1*-deficient [69]. In this important study, the presence of *BRCA1* gene expression was examined in 374 sporadic breast cancer patients. BRCA1 expression was lacking in 60.4% of the breast cancer patients. Patients that lost BRCA1 expression often had more advanced breast cancer and were tumor node metastasis stage III positive, lymph node positive and overexpressed c-Myc. The presence of hypermethylation of the *BRCA1* promoter region was examined. *BRCA1* promoter hypermethylation was observed in 16.4% of the breast cancer patients examined and was associated with BRCA1-, ER-, c-Myc overexpression, and the triple-negative phenotype. Thus loss of functional BRCA1 expression combined with increased expression of c-Myc was associated with a poor prognosis.

### Alteration of TP53 in Breast Cancer

Dysregulation of the *TP53* gene also occurs in sporadic breast cancer [70]. The genetic structure of the *TP53* gene was examined in 136 unselected sporadic breast cancer patients’ tumors. Approximately 40% of the tumor samples had *TP53* mutations. Moreover, this group postulated that these mutations were due to exposure of the breast cancer patients to environmental mutagens based on the frequency of G-T transversions and the incidence of guanosine mutations on the non-transcribed DNA strand of the *TP53* gene.

Polymorphisms of the *TP53* gene have been observed to have prognostic and predictive values in cancer therapy. The presence of two *TP53* gene polymorphisms, Arg72Pro and PIN3 (+16 bp) was observed in ninety-four women with sporadic breast cancer who were followed for a mean of 67.9 months after therapy [71]. This study determined that the different genotypes of the Arg72Pro and PIN3 (+16 bp) polymorphisms had no significant impact on survival in the sporadic breast cancer patients. However, this study observed that the patients which were treated with chemotherapy regmins without an anthracycline, that had the A2A2 genotype of the PIN3 (+16 bp) polymorphism, had a poorer overall survival than other genotypes. Although some controversity exists on use of anthracyclines in breast cancer patients, those with the A2A2 genotype of PIN3 (+16bp) polymorphism may benefit. These important studies document the importance of genetics in personalized medicine.

### Epigenetic Modification of the ER genes in Breast Cancer

Methylation of the promoter region of the *ER* and other genes has been associated with their decreased expression. The methylation status of the *ER*-alpha promoter region was examined in 138 sporadic breast cancers. The *ER*-alpha promoter region was observed to be methylated in 60.1% (83/138) tumors, including 57 of 69 of the tumors which did not express ER-alpha. This study determined that the probability of *ER*-alpha promoter methylation was increased in those cases that were ER-alpha- and PR- [72].

In a study of 100 sporadic primary breast cancers of which 51 were ER-alpha- and 49 ER-alpha+, ER methylation was observed in 98% of ER- and 65% of ER+ tumor samples. *ER-* promoter region methylation was also associated with lack of PR expression and double receptor negative expression status of the breast cancer specimens [73].

The methylation of the *ER*-beta promoter region was examined in 178 sporadic breast cancer patients. *ER*-beta promoter methylation was observed in 44.9% of breast tumor samples. In contrast *ER*-beta promoter hypermethylation was detected in only 14.3% of patients with benign breast hyperplasia. 58% of the ER-beta- tumors exhibited *ER*-beta promoter region methylation whereas 36.7% of the ER-beta+-positive cases exhibited methylation at the *ER*-beta promoter region. As the levels of *ER*-beta promoter methylation increased- the levels of ER-beta protein detected decreased in the tumor samples. A strong correlation between *ER*-alpha promoter methylation and *ER-*beta promoter methylation was observed [74].

### Expression of EGFR Family Members in Breast Cancer Patients

The EGFR family consists of four members. For the sake clarity in this review, we will refer to them as EGFR1 (a.k.a., EGFR, HER1, c-erbB1), HER2 (a.k.a., EGFR2, c-erbB2), EGFR3 (a.k.a., c-erbB3, HER3) and EGFR4 (a.k.a., c-erbB4, HER4). The expression of the EGFR1, HER2, EGFR3 and EGFR4 were examined by immunohistochemistry in 220 breast cancer carcinomas [75]. Increased expression of EGFR1 was detected in 16.4% of the tumors, increased expression of HER2 was observed in 22.8% of the tumors, increased expression of EGFR3 was detected in 17.5% of the tumors, and increased expression of EGFR4 was observed in 11.9% of tumors. Breast cancer patients with tumors that overexpressed EGFR1, HER2 or EGFR3 had reduced survival. In contrast, those breast cancer patients whose tumors displayed elevated levels EGFR4 had better survival than the breast cancer patient that expressed EGFR1, HER2 or EGFR3. Thus overexpression of EGFR1, HER2 and EGFR3 was association with a poor prognosis in breast cancer patients. In contrast, overexpression of EGFR4 is associated with a good prognosis. In addition, this group investigated the association of ER expression with the different EGFR molecules. Expression of EGFR1, HER2 and EGFR3 was associated with ER negativity in the breast cancer patients. Moreover those breast cancer patients that were ER+ and also EGFR1+, HER2+ or EGFR3+ had poorer survival than those breast cancer patients which were either ER+ and HER2+ or EGFR4+.

Additional studies were performed by the same group on the proliferative potential of the different types of EGFR-expressing cells [76]. These important studies suggest that EGFR1, HER2 and EGFR3 are linked with tumor proliferation, while EGFR4 did not appear to drive proliferation but may even play protective roles.

In another study, the expression of the EGFR1 and EGFR3 proteins were examined in 104 primary breast carcinomas comprising nine comedo ductal carcinoma *in situ* (DCIS), 91 invasive ductal carcinomas and four invasive lobular carcinoma by histochemistry. Increased expression of EGFR3 was observed in 67% of comedo DCIS, 52% of invasive ductal carcinomas, 71% of carcinomas containing both the *in situ* and invasive lesions and 25% of invasive lobular carcinomas [77]. 59% of ER- tumors, 63% of lymph node+ tumors and 63% of HER2 tumors were positive for EGFR3 expression. 67% of EGFR1+, 67% of HER2+ (67%), 75% TP53+ and 60% of cathepsin-D+ DCIS were positive for EGFR3 expression.

The expression of EGFR1, HER2, EGFR3 and EGFR4 was examined in 100 breast cancer patients [78]. By using immunohistochemistry techniques, 36% of the breast cancer samples were positive for EGFR1, 27%, of the breast cancer samples were positive of HER2, 26% of the breast cancer samples were positive for EGFR3 and 82% of the breast cancer samples were positive for EGFR4. The expression of these genes was also examined by RT-PCR and the similar results were observed. An association between decreased disease-free survival and expression of HER2 was observed. Co-expression of EGFR1 and HER2 was associated with a worse prognosis. In contrast, expression of EGFR4 was associated with a better outcome. EGFR4 expression appeared to antagonize the effects on HER2 on clinical outcome in the breast cancer patients which expressed both.

The link between ER-alpha and EGFR4 expression was examined in 103 breast cancer samples by both immunohistochemistry and RT-PCR [79]. In 25% of the breast cancer samples ER-alpha were not expressed. In addition, approximately 25% of them did not express EGFR4. About one-half of the ER-alpha- tumors did not express EGFR4 at both mRNA and protein levels. The luminal breast cancer cell lines MCF-7 and T47D cells expressed both ER-alpha and EGFR4. While the basal breast cancer line MDA-MB-231 and the HER2-overexpressing SK-BR-3 line expressed neither. These results have suggested roles for EGFR4 in ER-alpha mediated signal transduction in breast cancer.

The expression patterns of EGFR1, HER2, EGFR3 and EGFR4 were examined by real-time RT-PCR in 365 unselected primary breast cancers [80]. EGFR1 and HER2 were negatively associated with ER+ and PR+ breast cancers. In contrast, EGFR3 and EGFR4 were positively associated with ER+ and PR+ breast cancers. In a subsequent study with the ER-alpha+ breast cancer cell line MCF-7, beta-estradiol down regulated the expression of all four EGFR family member receptors as determined by RT-PCR. In contrast, the ER antagonist 4-hydroxy tamoxifen (4HT) inhibited the downregulation induced by beta-estradiol [81].

### Gatekeeper Mutations in HER2

**Figure 1:**
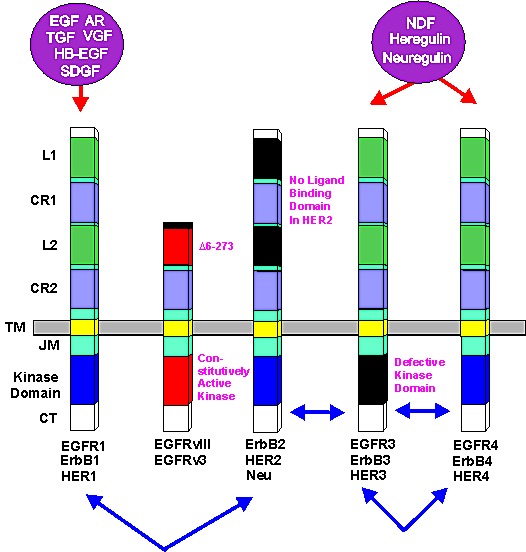
Epidermal Growth Factor Receptor Family Conserved domains of the four different EGFR family members are indicated by similar shading. L = ligand binding domain, CR = cysteine-rich domains. TM = transmembrane domain. CT = C-terminal domain which contains the phosphorylation sites. JM = juxta membrane domain. HER2 does not bind a ligand. The kinase domain in ERB3 is defective. Alternative names for each receptor are written underneath each receptor. Ligands which bind the receptors are indicated in purple circles above the receptors. Epidermal growth factor (EGF), vaccinia growth factor (VGF), amphiregulin (AR), heparin binding-EGF (HB-EGF), schwannoma-derived growth factor (SDGF), Neu differentiation factor (NDF), heregulin, neuregulin. Arrows between receptors indicate possible heterodimer formation between various EGFR family receptors.

The T798M mutation in *HER2* is considered a gatekeeper mutation as it affects the ability of small molecule inhibitors such as lapatinib to bind the ATP-binding pocket and the mutation confers resistance to the inhibitor [82]. In studies by a different research group which were aimed at determining the mechanism of resistance of breast cancer cells containing the *HER2* T798M mutation to lapatinib, it was shown that the cells overexpressed EGFR ligands [83]. In BT474 and MCF10A breast cancer cells transfected with the construct encoding *HER2*-T798M mutation, elevated HER2 kinase activity was detected and lapatinib did not block phosphorylation of HER2, EGFR3 or downstream Akt and ERK1/2. Increased levels of EGFR2 associated with PI3Kp85 were detected in the transfected cells and the BT474/*HER2*-T798M cells were also resistant to Herceptin. However these cells were sensitive to the pan-phosphoinositol-3-kinase (PI3K) inhibitors BKM120 and XL147 but not the mitogen-activated protein kinase kinase1/2 (MEK1/2) inhibitor CI-1040. The BT474/*HER2*-T798M transfected cells expressed elevated levels of the EGFR ligands EGF, TGF-alpha, amphiregulin (AR), and heparin binding EGF (HB-EGF). Thus targeting breast cancers with both HER2 inhibitors and PI3K inhibitors may be an appropriate technique to treat those breast cancer patients with *HER2*-T798M mutations [84]. Other growth factors also bind the EGFR family of receptors including: EGF, schwannoma-derived growth factor (SDGF), vaccinia growth factor (VGF), Neu differentiation factor (NDF) or neuregulins, and heregulin. A diagram of EGFR family members is presented in Figure 1.

### Roles of Aberrant mRNA Splicing in Sensitivity to Herceptin

The splice variant delete16*HER2*, which results from exon 16 skipping, has been shown to increase the transformation frequency of cancer cells. This mis-splicing results in resistance to Herceptin. In contrast, retention of intron 8 of the *HER2* gene after mRNA splicing results in the creation of herstatin which inhibits tumor cell proliferation. Likewise retention of intron 15 during splicing of *HER2* results in the p100 protein which also suppresses tumor cell proliferation [85].

### Mutations at the EGFR Gene Family in Breast Cancer

**Figure 2:**
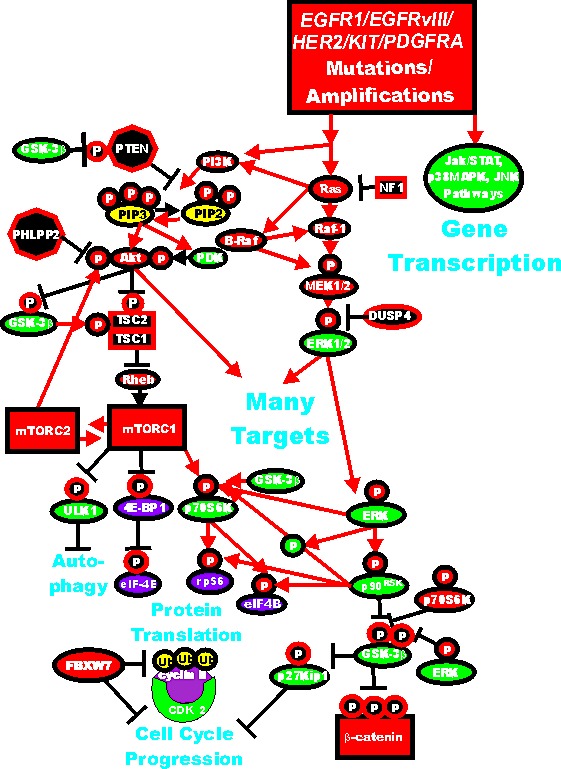
Dysregulated Expression of Upstream Receptors and Kinases Can Result in Activation of the Ras/Raf/MEK/ERK and Ras/PI3K/PTEN/Akt/mTOR and Other Signaling Pathways and Contribute to Malignant Transformation Sometimes dysregulated expression of growth factor receptors occurs by increased expression, genetic translocations or genomic amplifications which can lead to activation of the Ras/Raf/MEK/ERK, Ras/PI3K/PTEN/Akt/mTOR and other signaling pathways. Alternatively chromosomal translocations can occur in non-receptor kinases and other genes which result in activation of these pathways. Genes in the Ras/Raf/MEK/ERK and Ras/PI3K/PTEN/Akt/mTOR pathways that have activating mutations detected in human cancer and proliferative diseases are indicated in red ovals and squares. Tumor suppressor genes inactivated in certain cancer are indicated in black squares or octagons. Other key genes are indicated in green ovals. Red arrows indicate activating events in pathways. Blocked black arrows indicating inactivating events in pathways.

*HER2* is amplified in 20-25% of breast cancers. However, the roles of mutations/amplifications of other EGFR family members are not so clear. Mutations and amplifications of *EGFR1* have been detected in breast cancer [86, 87]. In a study consisting of 70 TNBC patient samples, mutations in the kinase domain were detected in 11% of the samples. This study amplified the region spanning exons 18 to 21 of the *EGFR1* gene. The investigators detected deletions in exon 19, which encodes part of the kinase domain. However, the authors indicated that these mutations appeared to be independent of the expression levels of the *EGFR1* protein that were detected by immunohistochemistry. A diagram illustrating some of the effects of mutations/amplifications in EGFR1/HER2 and other genes in breast and other cancers on signal transduction pathways is presented in Figure 2

In addition, amplification of the *EGFR1* gene has been detected in certain breast cancers [88]. The *EGFR1* gene was amplified in approximately 6% of the breast cancer patients they examined (n =175) and the samples displayed increased EGFR1 expression. They also examined patient samples which had amplified the *EGFR1* gene for hot-spot mutations in the *EGFR1* gene. No mutations were detected in exons 19 and 21.

The presence of *EGFR1* or *HER2* gene amplification or overexpression was investigated in a series of metaplastic breast carcinomas (MBC) [89]. MBC are basal like tumors that account for less than 1% of all invasive mammary carcinomas. Nineteen of the 25 (76%) MBC examined overexpressed EGFR1. *EGFR1* gene amplifications were detected in 37% of the tumors which overexpressed EGFR1. In contrast, only one case exhibited HER2 overexpression but *HER2* gene amplification was not detected in this study of MBC. The authors pointed out that some of the tumors that overexpressed EGFR1, but did not have *EGFR1* gene amplification, the expression of EGFR1 could have resulted from activating mutations in *EGFR1*, which resulted in its expression.

In a subsequent study by the same group, the presence of *EGFR1* amplification and activating mutations was examined in 47 MBC [90]. 32% of the samples exhibited overexpression of EGFR1. Within the subset that had EGFR overexpression, 34% had *EGFR1* gene amplification. This group examined these MBC samples for the presence of activating mutations in exons 18, 19, 20, and 21 of the *EGFR1* gene. No activating mutations of *EGFR1* in exons 18, 19, 20 and 21 were detected in these MBC patient samples.

The epidermal growth factor receptor variant III (*EGFRvIII*) is a genetic truncation of the *EGFR1* gene. It encodes a constitutively-active truncated EGFR1 protein which has been implication in many types of cancers, (e.g., brain, breast, prostate and others) [91-103].

Introduction of a construct encoding *EGFRvIII* into MCF-7 breast cancer cells resulted in HER2 phosphorylation, which the authors suggested occurred through heterodimerization and cross-talk [91]. The MCF-7/EGFRvIII transfectants had approximately a 3-fold increase in colony formation. MCF-7/EGFRvIII were more tumorigenic that MCF-7 cells in athymic nude

The EGFRvIII protein is detected in various human cancers, but not in normal tissues. The presence of EGFRvIII mRNA was examined in primary invasive breast cancer by utilizing laser capture microdissection (LCM)/RT-PCR. A high incidence (67.8%) of EGFRvIII transcripts was observed in pure breast cancer cells [92]. Furthermore, 57.1% of the infiltrating breast carcinomas expressed both EGFR1 WT and EGFRvIII mRNA in the same tumor. In contrast, no detectable EGFRvIII mRNA was seen in samples of normal breast tissue. These expression results were further confirmed by immunohistochemical analysis. Thus there was co-expression of EGFRvIII and EGFR WT in some human invasive breast cancer tissue but not in normal breast samples. It must be pointed out that other studies consisting of 55 breast cancer cell lines and 170 primary breast cancer did not observe similar results and the authors concluded that expression of EGFRvIII is extremely rare in breast cancer [93]. However, in a study by a different research group, the expression of EGFRvIII mRNA in women with breast cancer was examined by an RT-nested PCR. EGFRvIII mRNA was detected in the peripheral blood of 30% of 33 low risk, early stage patients, in 56% of 18 patients selected for neoadjuvant chemotherapy, in 63.6% of 11 patients with disseminated disease but not in any of 40 control women [94]. Interesting in the low risk, early stage patients, EGFRvIII expression was associated with ER- or HER2+. In another study of 225 breast cancer patients, the expression of EGFR1, phosphorylated EGFR1, and EGFRvIII, was examined by immunohistochemistry and the patient outcomes were also followed [95]. 48% of the patients displayed EGFR1, 54% of the patients were positive for phospho-EGFR, and 4% of the patients were positive for EGFRvIII. EGFR1 expression correlated with negative hormone receptor status, worse relapse-free survival and overall survival than those patients with did not express detectable EGFR1 [95]. Interestingly there did not appear to be any association between expression of phospho-EGFR or EGFRvIII expression with clinical outcome. This study indicated that prognostic value of EGFR1 expression was most important in the HER2+ and the ER-/PR- subgroups.

EGFRvIII expression may down regulate PR expression in certain luminal B tumors [96]. These tumors are ER+ but are characterized as having an aggressive behavior which is 4HT resistant. This subset of breast cancers displays increased EGFR1, HER2 and downstream PI3K/PTEN/Akt/mTORC1 pathway activation [96,97].

EGFRvIII interacts with HER2 [98] and chemokine (C-X-C motif) receptor 4 (CXCR4) [99,100] to activate signaling pathways important in migration, invasion and tumorigenesis. These interactions between EGFRvIII and HER2 and CXCR4 may be prolonged in comparison to interactions between EGFR1 and HER2 and CXCR4 as it is more difficult to down regulate the constitutive nature of EGFRvIII. CXCR4 is highly expressed in breast cancers and implicated in metastasis [101] and cancer initiating cells (CICs) [102].

Recently EGFRvIII has been implicated in primary breast cancers and breast CICs. Its expression has been associated with the Wnt/beta-catenin pathway and downstream beta-catenin target gene expression and the expression of genes associated with self-renewal. Its expression has been linked with increased *in vitro* mammosphere formation and tumor formation [103].

HER2 normally has to heterodimerize with another EGFR family member for activity. However, if the *HER2* gene is amplified, HER2 activity is induced and the abnormal breast tumor growth is dependent on HER2 activity for growth [104]. An activating mutation has been detected in HER2+ lung cancer patients in the germline from a Japanese cancer patient with a familiar history of lung cancer as well as sporadic cancer patients [105]. The mutation occurred in the transmembrane region and may be responsible for increased stability and dimerization. This mutation activated downstream HER2 signaling molecules including Akt and p38^MAPK^.

Somatic mutations in the kinase domain of the *HER2* gene, which result in its activation have been detected in lung cancer patients [106]. In a study involving non small cell lung cancers (NSCLC), *HER2* mutations were detected in 1.6% (11 of 671) of NSCLC cancer specimens examined but were absent in other types of cancers. The *HER2* mutations were in-frame insertions in exon 20 corresponding to a similar region as in the *EGFR* gene where** insertions were detected in NSCLC patients. Interestingly the *HER2* mutations were more frequent in the lung cancer patients that never smoked. *HER2* mutations were detected more frequently in patients of Oriental ethnicity than other ethnicities. The mutations were also more frequently detected in females.

Recently mutations in *HER2* have been detected in breast cancer patient samples which lack *HER2* gene amplification. Thirteen *HER2* mutations were characterized from twenty-five patient samples which had *HER2* mutations but lacked *HER2* gene amplification. 7 mutations were activating and resulted from point mutations and in-frame deletions. Some mutations (L755S) resulted in lapatinib resistance; however this was not an activating mutation. All of the cells containing the *HER2* mutations were sensitive to the irreversible HER2 kinase inhibitor, neratinib [107].

Loss of HER2 activity also results in loss of phosphorylated EGFR3. It has been shown that HER2 can dimerize with EGFR3 to drive breast cancer proliferation [104]. Loss of EGFR3 activity in HER2+ breast cancer cells inhibited their growth. This growth inhibition mediated by loss of HER2 or EGFR3 and could be overcome by introduction of a construct encoding activated Akt. Importantly this group demonstrated that a key function of EGFR3 was to couple the response of HER2 to the PI3K/PTEN/Akt/mTORC pathway.

Less is known about the roles of EGFR3 and EGFR4 in breast cancer [108]. EGFR3 is naturally kinase-inactive. However it interacts with the EGFR family members and serves to transduce signals to the PI3K/PTEN/Akt/mTORC1 pathway. Since EGFR3 is kinase-inactive, it will be more difficult to isolate inhibitors specific to it. However, it is still important and it can serve in pathways responsible for drug resistance [109].

The functions of EGFR4 in breast and other cancers have been investigated and recently summarized [110]. Similar to the *EGFR1* gene, *EGFR4* encodes a protein contains an extracellular ligand-binding domain, a hydrophobic transmembrane domain, an intracellular tyrosine kinase domain, and carboxyl-terminal tyrosine residues [110,111]. These carboxyl-terminal tyrosine residues are phosphorylated after ligand binding and allows coupling of the receptor with other signaling molecules [111]. Ligand binding to EGFR4 stimulates either homodimerization or heterodimerization of EGFR4 with another EGFR family member. Activation of EGFR4 signaling results in cleavage and release of the EGFR4 cytoplasmic domain from the membrane, which then may traffic to the nucleus and mitochondria to exert additional biological effects [110,112,113].

The roles of EGFR4 in cancer are complex. In some cancers (larynx, pancreatic and prostate) EGFR4 may function as a tumor suppressor [110,114-116]. EGFR4 expression in breast, cervical and ovarian cancers is associated with a favorable prognosis [110, 113,117,118]. The *EGFR4* Q646C mutant results in an EGFR4 protein which undergoes ligand-independent homodimerization and tyrosine phosphorylation which interestingly suppresses colony formation of breast, pancreatic and prostate cell lines [110, 119-121]. Introduction of the constitutively active *EGFR4* I658Q mutant into breast, ovarian and prostate cell lines induces apoptosis [122].

However, in some cell types and biological situations EGFR4 has oncogenic activities [120]. EGFR4 is overexpressed in certain cancers including ependymomas and medulloblastomas [111,116,123]. Overexpression of EGFR4 in lung carcinomas results in increased proliferation [116.^123^]. Furthermore, overexpression of HER2, EGFR4, and the EGFR4 ligand NRG1-beta in medulloblastoma results in increased metastasis [124]. Likewise over-expression of EGFR4 in conjunction with EGFR1 and HER2 results in breast cancers correlates with poor prognosis [125]. In contrast overexpression of EGFR4 by itself results in a more favorable outcome.

Silencing endogenous EGFR4 expression in ER+ MCF7 and T47D breast tumor cell lines reduces anchorage-independent proliferation stimulated by an EGFR4 ligand such as neuregulin-2beta [110, 126]. HER2 tyrosine kinase activity, rather than EGFR4 tyrosine kinase activity was required for neuregulin-2beta to stimulate cell proliferation [110]. Interesting the sites of EGFR4 tyrosine phosphorylation, but not the sites of HER2 phosphorylation, were required for neuregulin-2beta to regulate cell proliferation [110]. Thus the roles of EGFR4 in cancer remain complex and are influenced by the expression of other EGFR family members.

### Neurofibromin 1 and GTPase Activating Proteins (GAP)

Neurofibromin 1 (*NF1*) is a tumor suppressor gene. It encodes a GTPase which normally serves to regulate Ras signaling. It is mutated in neurofibromatosis patients. These patients have elevated (constitutive) Ras activation. The tumor suppressor *NF1* has been implicated in sporadic breast cancer. Recent studies have suggested that *NF1* is a breast cancer driver gene. *NF1* is deleted or mutated in 27.7% of all breast carcinomas [127]. In a study of inbred Chaos3 mice, investigators noted high levels of genetic instability which can lead to mammary tumors. The genomically-characterized mammary adenocarcinomas from these mice displayed deletions of certain genes, and the *NF1* gene was deleted in the vast majority of the mouse mammary adenocarcinomas. These mammary adenocarcinomas exhibited constitutive Ras hyperactivation and sensitivity to Ras pathway inhibitors.

Also alternative mRNA splicing events can give rise to different NF1 isoforms. NF1 and Ras expression were examined in 22 sporadic breast cancers, 18 benign lesions and 6 normal breast tissues by tissue microarrays. NF1 and CELF3-6 RNA expression was examined by RT-PCR in the breast samples. NF1 and Ras expression displayed no difference in expression when examined by immunohistochemistry assays. In contrast, NF1 isoforms were determined to shift from the type II mRNA isoform in normal breast in normal breast cancer to the type I mRNA isoform in breast carcinoma. The authors did not detect a shift in CELF mRNA cofactor expression that was related to the shift in NF1 mRNA isoforms. The authors suggest that there is a NF1 isoform shift in expression from type II to type I which could be important in the development and progression of sporadic breast cancer [128].

NF1 normally serves to regulate Ras and thus is implicated in the regulation of both the PI3K/PTEN/Akt/mTORC1 and Raf/MEK/ERK pathways. Successful targeting of Ras may improve the therapy of patients with *NF1* mutations. Alternatively, these patients may be sensitive to combined treatment with MEK and PI3K pathway inhibitors.

Genes that serve to regulate the Ras gene may be tumor suppressors as when they are mutated the Ras pathway is turned on. The RasGAP2 gene (*RASAL2*) is one such gene, it is a tumor and metastasis suppressor which is mutated or suppressed in breast cancer. Inactivation of RASAL2 was shown to be associated with tumor growth, progression and metastasis in animal models. In human *RASAL2* loss was associated with metastatic disease and decreased expression of RASAL2 was associated with the recurrence of luminal B breast tumors [129].

The methylation state of the Ras association domain-containing protein 1 (*RASSF1A*) gene was examined in 36 breast cancer patients in breast cancer tissue as well as their adjacent normal tissues [130]. *RASSF1A* may function as a tumor suppressor gene. Methylation of the *RASSF1A* gene was detected in 61.1% of the breast tissues but not in their normal adjacent tissues. The methylation of the *RASSF1A* gene resulted in deceases in mRNA and protein levels of 33.3 and 44.4% respectively. In contrast, the RASSF1A protein was detected in normal tissues. Methylation of the *RASSF1A* gene did not appear to be associated with clinical parameters, such as age, histological types, TNM stages and lymph node metastases.

### RAS Gene Family Mutations/Alterations in Breast Cancer

In a genetic study examining the mutational status of the Ras and PI3K pathway genes in 40 breast cancer cell lines, mutations were detected at approximately 25% in Ras pathway members (*KRAS*, *HRAS*, *NRAS*, and *BRAF*) and 54% of PI3K pathway members (*PTEN*, *PIK3CA*). However, unlike the mutational status of these two families in colo-rectal cancer, this study did not detect mutation in both pathway family genes in single cell lines very frequently [131]. In subsequent studies by the same group with 41 breast cancer cell lines, this group found 146 mutations among twenty-seven cancer causing genes, which resulted in an average of 3.6 mutations per cell line. Mutations in TP53, RB and PI3K pathways were frequently detected in the breast cancer cell lines. Importantly these investigators could identify mutational profiles that were associated with luminal-type and basal-type breast cancer cell lines. The luminal mutational profile included E-cadherin (*CDH1*) and mitogen-activated protein kinase kinase 4 (*MAP2K4* a.k.a. MEK4) genes and amplifications of the cyclin D1, (*CCND1*), *HER2* and mouse double minute 2 homolog (*MDM2*), while the basal mutational profile included: *BRCA1*, *RB1*, *RAS* and *BRAF* gene mutations and deletions of tumor suppressors p16 (*CDKN2A*) and p14ARF (*CDKN2A* a.k.a. *INK4A*) [132].

Activation of the Ras pathway is often detected in breast cancer in the absence of mutations of the *RAS* genes [133]. Gene expression signature analysis has revealed that Ras pathway dependence can predict the sensitivity to inhibitors targeting the Raf/MEK/ERK and PI3K/PTEN/Akt/mTORC1 pathways. The Ras pathway activation is associated with sensitivity to MEK inhibitors but resistance to Akt inhibitors in breast and lung tumors. The Ras pathway signature is a better indicator of Ras pathway dependency than mutations at *KRAS*, as there can be many genetic mutations which can result in Ras pathway dependency (*e.g*., upstream receptors, *EGFR1* and others as well as mutations in *BRAF* and other downstream signaling molecules). The Ras pathway signature was determined to be activated in breast cancers which were sensitive to MEK inhibitors but resistant to Akt inhibitors. The Ras pathway signature was shown to be increased in ER- breast cancers and lung adenocarcinomas. The Ras pathway signature also predicts resistant to the EGFR1-targeting agent cetuximab (Erbitux) in metastatic colorectal cancer [134].

Upon novel global gene expression profiling on 47,293 gene transcripts in 128 invasive breast cancers, the Ras-like, estrogen-regulated, growth-inhibitor (*RERG*) gene was determined to be a key marker of the luminal BC class and could be used to separate distinct prognostic subgroups [135]. These observations were further explored by performing immunohistochemistry on tissue microarrays containing 1,140 invasive breast cancers [135]. These results showed that the RERG gene is one of the highest ranked genes to differentiate between ER+ luminal and ER- non-luminal cancers. RERG expression was positively associated with the following markers of luminal differentiation: ER+, the cytokeratins (CK7/8, CK18 and CK19) and FOXOA1. RERG expression was also associated with other markers of good prognosis namely, small size, lower histologic grade an d AR, BRCA1, fragile histidine triad protein (FHIT, Bis(5’-adenosyl)-triphosphatase), and p21^Waf-1^ and p27^Kip-1^ and inversely associated with the proliferation markers MIB1 (a monoclonal antibody which is directed to a different epitope on the Ki67 protein than the original Ki67 antibody) and TP53. Importantly RERG expression was associated with longer survival.

The Rab-coupling protein RCP (a.k.a RAB11FIP1), is located at a chromosomal region frequently amplified in breast cancer. Introduction of constructs encoding RCP into normal human mammary epithelial cells MCF-10A cells lead to cells with tumorigenic properties. Likewise knock-down of RCP in breast cancer cell lines decreased the tumorigenic properties of the cells. Therefore RCP is a frequently amplified gene in breast cancer and suggest roles for the Rab family in carcinogenesis [136].

### Interactions between Ras and Bmi-1 in Breast Cancer

The B-lymphoma Moloney murine leukemia virus insertion region-1 (Bmi-1) gene functions in stem cell maintenance. It is a member of the polycomb group of transcription repressors. Bmi-1 exerts it effects by suppression of the p16Ink4A/ARF tumor suppressor. Increased expression of Bmi-1 is detected in many cancers. The effects on Bmi-1 overexpression were examined on MCF-10A cells. While Bmi-1 overexpression by itself did not result in the oncogenic transformation of MCF-10A cell, co-expression of activated H-Ras (RasG12) resulted in the oncogenic transformation of MCF-10A cells. The Bmi-1/H-Ras transformed cells exhibited properties of cells that had undergone the epithelial to mesenchymal transition (EMT). Bmi-1 inhibited senescence and allowed the proliferation of cells expressing high levels of activated H-Ras [137]. Subsequent studies by the same group have indicated that knock-down of Bmi-1 in breast cancer cell lines decreased their aggressive nature *in vivo* in tumor transplant studies [138].

### Mutations in Components of the PI3K/PTEN/Akt mTOR Pathway in Breast Cancer

In a study which examined MBC, *PIK3CA* mutations were detected in 47.4% of the cancers which were aggressive and also chemoresistant. In contrast, *PI3KCA* mutations were detected in 34.5% of hormone receptor-positive cancers, 22.7% of 75 HER2-positive cancers, 8.3% of basal-like cancers and none of claudin-low tumors examined in this study. MBCs and claudin-low breast cancer subsets displayed enrichment for markers linked to stem cell function and EMT. It was postulated that MBCs and claudin-low tumors are enriched with CICs and may arise from an earlier, more chemoresistant breast epithelial precursor than either basal-like or luminal cancers. However in this study, no mutations at *PIK3CA* were detected in the claudin-low cancers, so the roles of *PIK3CA* mutations in these cancers are not clear [139].

In a study with 547 human breast cancer patient samples and 41 established cell lines, the mutational status of *PIK3CA*, *AKT* and *PTEN* were analyzed as well as the effects of pathway mutations on the sensitivity to PI3K inhibitor LY294002 [140]. *PIK3CA* mutations were determined to be more frequent in ER+ (34.5%) and HER2+ (22.7%) than in basal-like tumors (8.3%). *AKT1* (1.4%) and *PTEN* (2.3%) mutations were determined to be restricted to ER+ breast cancers. Interestingly cells with *PIK3CA* mutations were less sensitive to the PI3K inhibitor LY294002 inhibitor than breast cancer cells which had loss of PTEN activity. Thus, PI3K pathway aberrations likely play a distinct role in the pathogenesis of different breast cancer subtypes. The specific aberration present may have implications for the selection of PI3K-targeted therapies in hormone receptor-positive breast cancer.

In a study which examined the genetic structure of the *PIK3CA* gene in 452 breast cancer patients, *PIK3CA* mutations were observed in 33.4% of the breast cancer patients [141]. *PIK3CA* mutations were more frequently detected in ERalpha+ and PR+ breast cancers (41.1%), than in TNBCs (ER-,PR-, HER2-) (12.5%.). Patients which had *PIK3CA* mutations had a longer metastasis-free survival period than the overall population.

The mutational status of the *PIK3CA* gene was examined in eighty HER2+ breast cancer patients as well as clinical outcome of the patients after Herceptin treatment [142]. The *PIK3CA* gene was determined to be mutated in 21.3% of HER2+ breast cancer patients that had been treated with herceptin for one year. Improved disease free survival was observed in those patients with WT *PIK3CA* as opposed to those patients with mutant *PIK3CA*. The *PIK3CA* gene is also mutated in some ovarian cancer patients [143].

PI3K-p110 (*PIK3CA*) protein expression was examined in 1,394 early stage breast cancer samples. Elevated PI3K-p110 was associated with the basal-like breast cancers, HER2+ breast cancer, and triple negative non-basal breast cancers. In contrast, the luminal class of breast cancers had reduced levels of PI3K-p110 in comparison to the other classes. PI3K-p110+ breast cancer patients had shorter disease free survival. Thus these studies demonstrated that PI3K-p110 expression is a biomarker associated with poor prognosis in breast cancer [144].

### Interactions between PIK3CA Mutations and HER2 Amplification in Breast Cancer

Introduction of genetic constructs containing *PIK3CA* mutations E545K and H1047R into MCF-10A human mammary epithelial cells that also overexpress *HER2* conferred enhanced growth properties to MCF10A/HER2 cells in comparison to cells which lacked the introduced mutant *PIK3CA* gene [145]. Upon introduction of mutant *PIK3CA* (H1047) into MCF-10A cells which overexpress HER2, the expression of EGFR3/EGFR4 ligand heregulin (HRG) was detected. In contrast, introduction of mutant *PIK3CA* (E545K) gene into the MCF-10A cells which overexpressed HER2 did not result in the expression of HRG. Silencing HRG with siRNA inhibited the growth of MCF-10A/*HER2/PIK3CA*-H1047R cells but not MCF-10A/*HER2/PIK3CA*-E545K-expressing cells. The HRG siRNA synergized with the HER2 inhibitors herceptin (trastuzumab) and lapatinib. Treatment of the cells with a PI3K inhibitor (BEZ235) suppressed HRG and P-AKT levels. When the cells were cotreated with BEZ235 and lapatinib, a complete suppression of growth was observed. These important result document interactions between HER2 and certain *PIK3CA* mutations and point to the possible co-targeting of HER2 and PI3K in certain breast cancers.

Deregulation of the PI3K/PTEN/Akt/mTORC1 pathway by gene mutations has been estimated to occur in >70% of breast cancers [146]. In ER+ breast cancers, activation of the PI3K/PTEN/Akt/mTORC1 pathway can result in both estrogen-dependent and estrogen-independent ER activity and contribute to estrogen-independence and potentially loss of sensitivity to hormonal based therapies. Activation of the PI3K/PTEN/Akt/mTORC1 pathway can also result in resistance to HER2 inhibitors in HER2+ cells. Inhibition of the PI3K/PTEN/Akt/mTORC1 pathway can overcome resistant to hormonal and anti-HER2 targeted therapies [146]. Encouraging results have been obtained in breast cancer clinical trials with combinations of various inhibitors. These results predict that combinations of HER2, PI3K, mTORC2 inhibitors and hormonal based therapeutics may be appropriate for the treatment of certain breast cancer patients which are resistant to current therapies.

The transforming activity of mutant PI3K has been linked to its ability to bind phosphorylated YXXM motifs present in activated receptor tyrosine kinases (RTK) or adaptor molecules, potentially in association with the PI3K-regulatory subunit, p85. EGFR3 is a potent activator of the PI3K/PTEN/Akt/mTORC1 pathway. The requirement for EGFR3 in PI3K-mediated transformation of mammary epithelium was investigated [147]. Conditional loss of *EGFR3* in mammary epithelium lead to a delay in PI3K-H1047R-mediated mammary hyperplasia. However, these studies demonstrated that tumor latency and PI3K signaling were not perturbed. In the *EGFR3*-deficient mammary tumors, the PI3K-H1407R protein was determined to be associated with several tyrosyl phosphoproteins. These studies also demonstrated that inhibition of other EGFR family members with lapatinib did not inhibit the mutant PI3K-mediated signaling. However, co-inhibition of PI3K with the PI3K-alpha specific inhibitor BYL719 and EGFR-mediated signaling with lapatinib was more effective in suppressing growth than treatment with either the PI3K-alpha inhibitor or lapatinib alone. Additional studies by this group determined that co-inhibition of PI3K and EGFR suppressed growth and PI3K signaling in human breast cancer cells containing the mutant *PIK3CA*-H1047R gene. These studies point to the possibility of co-targeting of PI3K and EGFR in certain breast cancers.

### Mutations at PIK3CA and PTEN Can Confer Resistance to Herceptin in HER2+ Breast Cancer

An RNA interference screen was performed to identify some of the genes involved in resistance to herceptin. PTEN was identified as a modulator of resistance to Herceptin in cell cultures experiments. Likewise oncogenic *PIK3CA* mutations would confer resistance to Herceptin in cell cultures [148]. In a screening of samples from 55 breast cancer patients, *PIK3CA* mutations or low expression of PTEN was associated with resistance to Herceptin.

### Deregulation of PI3K and PTEN in Metastatic Breast Cancer

The mutational status of the *PIK3CA* gene and expression of PTEN was examined in breast cancer specimens that differed in the state of malignancy (e.g., primary vs. metastatic breast cancer from the same cancer patient) [149]. The *PIK3CA* gene was determined to be mutated in 19 (40%) of primary tumors and 21 (42%) of metastatic cancers. PTEN expression was examined by immunohistochemistry and PTEN expression was lost in 14 (30%) primary tumors and 13 (25%) metastases. Thus the *PI3KCA* gene is frequently mutated and PTEN expression is often lost in breast cancer.

### Epigenetic Regulation of Additional and Novel Genes Associated with Breast Cancer

Epigenetic profiling has recently resulted in the identification of genes associated with breast cancer tumorigenicity that were methylated [150]. This study identified 264 hypermethylated loci in genomic CpG islands. Hierarchical clustering in terms of the levels of methylation at the loci resulted in generation of at least three distinct groups of breast cancer patients. Namely the methylation levels were distributed into three different groups, ER+/PR+ breast cancer patients, time to tumor relapse and lymph note metastasis. Methylation of six genes (*RECK*, *SFRP2*, *UAP1L1*, *ACADL*, *ITR*, and *UGT3A1*) was associated with decreased relapse free survival. Reversion-inducing-cysteine-rich protein with kazal motifs (*RECK*) is thought to be a metastasis-suppressor gene and may interact negatively with matrix metalloproteinase (MMP9). Secreted frizzled-related protein 2 (SFRP2) is a soluble modulator of Wnt signaling. Methylation of this gene has been association with breast and other cancers [151]. *UAP1L1* encodes a UDP-N- acteylglucosamine pyrophosphorylase 1-like 1 protein. *ACADL* is a member of the acyl-CoA dehydrogenase family. This is a family of mitochondrial flavoenzymes involved in fatty acid and branched chain amino-acid metabolism.** The *ACADL* gene product is associated with long-chain 3-hydroxyacyl-coenzyme A dehydrogenase deficiency. The *ITR* gene (ITR/GPR180) is a G protein-coupled receptor). The *UGT3A1* gene is a member of the** UDP glycosyltransferase 3 family.

### Drug Resistance and CICs

Drug resistance breast cancer cells are enriched in populations of cells which have characteristics of cancer stem cells. These cancer cells with stem like characteristics are referred to cancer initiating cells (CICs) [152-175]. In the study by Britton and colleagues with both clinical fine needle aspirates obtained from breast cancer patients as well as established breast cancer cell lines, the expression of ATP-binding cassette sub-family G member 2 (ABCG2=BCRP1) was monitored [175]. In the studies with the side populations from MCF-7 and MDA-MB-231 cells, increased ABCG2 was observed in the side populations as well as elevated resistance to the chemotherapeutic drug mitoxantrone. The increase in the side populations may be due to increased drug transporter expression in the cells with the CIC phenotype as the drug transporter would exclude the drugs from the cells. The presence of the side populations in the fine needle aspirates was associated with ER-negative breast cancers and TNBCs which also had elevated ABCG2 protein expression. Breast CICs can be characterized by increased expression of CD44 and decreased expression of CD24 (CD44↑/CD24↓) compared to the remaining cancer cells which are referred to as the bulk cancer cells (BCs). The CICs and BCs differ in their gene expression patterns which may have resulted from epigenetic and other mechanisms [176]. Thus the CICs and BCs will likely have different signaling pathways activated/suppressed which will require different therapeutic approaches to eliminate both populations of cancer cells.

### Involvement of HER2 in Breast CICs

HER2 is expressed in the CIC population [177, 178]. The expression of HER2 is modulated by the tumor microenvironment. Targeting of HER2 in these CICs may be an appropriate therapeutic approach. Interestingly herceptin suppressed tumor growth of the CIC in mouse xenograft models but in not established breast tumors [176].

Initially was thought that herceptin would only be effective in breast cancer patients which overexpressed HER2, some clinical studies have shown that herceptin may target breast cells which did not overexpress HER2 [179]. These results have led to the hypothesis that HER2 is an important molecule expressed on breast CICs and further studies have suggested that HER2 expression may be induced by signals in the microenvironment in breast CICs which lack HER2 gene amplification.The effectiveness of herceptin is thought to be due to its ability to target the breast CIC population as well as the PI3K/PTEN/Akt/mTORC and other signaling pathways [176, 177, 180].

Herceptin may also be effective in the treatment of breast cancer patients which lack amplification of *HER2*, documenting a role for HER2 in the growth of these normally *HER2*-negative cancers [181]. These authors demonstrated that HER2 was expressed in ER+, HER2- luminal breast cancers and regulates the self renewal of the CIC sub-population. HER2 expression was not due to gene amplification but was determined to result from receptor activation of NF-kappaB (RANK)-ligand in the bone microenvironment.

### PI3K Pathway in Breast CICs

The *PIK3CA* gene may be mutated in some breast CICs [182]. The PI3K/PTEN/Akt/mTORC pathway has been reported to be important in breast CICs. Side-population positive MCF-7 breast cancer cells [MCF-7/(CIC)] were isolated from parent MCF-7 bulk cancer [MCF-7/(BC)] [183]. The MCF-7/(CIC) cells displayed increased drug transporter activity and enhanced colony-formation ability *in vitro* and greater tumorigenicity *in vivo* than the MCF-7/(BC). The expression of critical pathways were compared between MCF-7/(CIC) and MCF-7/(BC). The PI3K/PTEN/Akt/mTORC1 and STAT3 pathways were shown to be differentially expressed in the MCF-7/(CIC) population and responsible in part for their enhanced survival [183].

### Deregulation of Akt in Breast Cancer

Mutations of Akt are relatively rare in breast cancer, however, aberrant activation of either upstream *PIK3CA* or polymorphism of the PH domain and leucine rich repeat protein phosphatase 2 (*PHLPP2*) gene can result in Akt expression. In a study which examined the mutational status and polymorphism of genes in the PI3K/PTEN/Akt/mTORC1 pathway, DNA was isolated from fine needle aspirations of 267 stage I-III breast cancers [184]. In this study, 28 genes were examined for 163 known cancer-related DNA sequence variations by Sequenom technology. The PI3K pathway was determined to be frequently altered in breast cancers as at least one mutation in 38 alleles corresponding to 15 genes in 108 (40%) of the breast cancer samples. The *PIK3CA* gene was determined to be the most frequently mutated (16.1% of all samples), the F-box and WD repeat domain containing 7, E3 ubiquitin protein ligase (*FBXW7*) gene, the second (8%), the *BRAF* gene, the third (3.0%), the *EGFR1* gene, the fourth (2.6%), the *AKT1* and *CTNNB1* genes (beta-catenin) the fifth and sixth (1.9% each), the *KIT* and *KRAS* genes (1.5% each), and the *PDGFRA* gene, the seventh (1.1%). Polymorphism at the *PHLPP2* phosphatase which activates Akt was observed in 13.5% of the patient samples. *PIK3CA* mutations were observed more frequently in ER+ cancers compared to TNBC (19 vs. 8%). Interesting a high frequency of *PIK3CA* mutations (28%) was observed in HER2+ breast tumors. In TNBC, *FBXW7* mutations were significantly more frequent compared to ER+ tumors (13 vs. 5%). FBXWZ is a component of ubiquitin ligase (SKP-cullin-F-box) and may bind cyclin E and target it for ubiquitin-mediated degradation (Figure 2).

Some investigators have suggested that other molecules besides Akt are important in breast cancer and have proposed Akt-independent signaling mechanisms [185]. This group demonstrated that in some breast tumors cultured in an anchorage-independent fashion displayed minimal Akt activation and decreased reliance on Akt for growth. In contrast, these cells had strong PDK1 activation and membrane localization and were dependent on the activated PDK1 substrate serum/glucocorticoid regulated kinase family, member 3 (SGK3), which is related in structure to Akt. SGK3 has been shown in additional studies to be linked with breast cancer and is induced by estrogen in breast cancer cells and is associated with ER expression [186]. Like Akt, SGK3 can phosphorylate GSK-3beta and TSC-2 which results in their inactivation and activation of mTORC1 and stimulation of protein translation [187, 188]. Knowledge of the particular kinase or other type of protein, responsible for the malignant potential of breast cancer cells could aid therapy by the use of more effective inhibitors which target the particular enzyme affected and responsible for the abnormal growth.

### Activated Akt as a Marker for Sensitivity to Drug Therapy

Phosphorylation (activation) of Akt has been shown to predict the effectiveness of paclitaxel chemotherapy in node-positive breast cancer patients [189]. In the National Surgical Adjuvant Breast and Bowel Project (NSABP) B-28 trial, the effectiveness of adding paclitaxel to doxorubicin (a.k.a Adriamycin) plus cyclophosphamide (AC) was examined in breast cancer patients (median follow up 9.1 years). Enhanced effectiveness of adding paclitaxel to AC was observed in those breast cancer patients who expressed elevated P-Akt. In contrast, no enhanced effectiveness of adding paclitaxel to AC was detected in the breast cancer patients who did not express activated P-Akt. Thus addition of paclitaxel to breast cancer patients which are P-Akt- does not appear to increase the effect of AC therapy, while addition of paclitaxel to those breast cancer patients which are P-Akt+ does appear to improve therapy.

### PTEN and Cytokines and their Involvement in HER2-Resistance and CIC Survival

PTEN is important in breast CIC survival. Knockdown of PTEN expression was shown to result in increases in normal and malignant human mammary stem/progenitor cells both *in vitro* and *in vivo*. This increase in progenitor cells was mediated by increased Akt activation which resulted in the phosphorylation of GSK-3beta which in turn led to activation of the Wnt/beta-catenin pathway. The increases in progenitor cells could be suppressed by the Akt inhibitor perifosine [190]. PTEN is also important in the resistance of breast cancers to herceptin and other therapeutic approaches [191].

**Figure 3:**
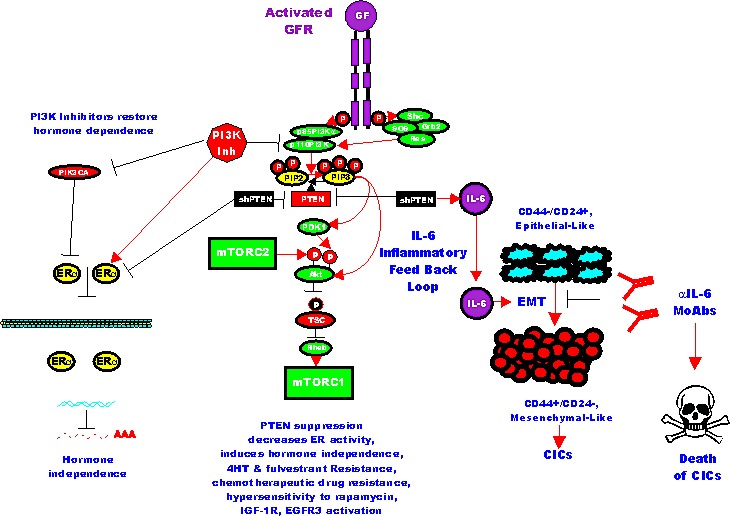
Effects of Targeting PI3K/PTEN/Akt/mTORC1 and IL-6 on Breast Cancer Hormonal Dependency, EMT and CICs Mutations at PIK3CA can alter hormonal dependency. Silencing PI3K with PI3K inhibitors can restore hormonal dependency. Silencing PTEN can also result in hormonal-independenc, 4HT and fulvestrant resistance. Silencing PTEN can in some cases also lead in some cases to IL-6 production and an IL-6 inflammatory feed back loop which results in EMT and CIC formation. Silencing of IL-6 with monoclonal antibodies (MoAb) can prevent this loop and result in the death of the breast CICs.

Interleukin-6 (IL-6) is cytokine which is an important immune-regulator. IL-6 is also important in HER2-resistance as it can expand the CIC population [192-194]. Decreases in PTEN expression has been implicated in herceptin-resistance. In HER2+ cell lines generated by knocking down PTEN, which were resistant to herceptin, the resistance was shown to be due to activation of an IL-6 inflammatory feed back loop. This IL-6 inflammatory loop resulted in the expansion of breast CIC which have an EMT phenotype and secrete 100-fold more IL-6 than in the parental cells which did not have PTEN knocked down. The authors of this important study also determined than treatment with an IL-6R Ab inhibited this IL-6 regulatory loop and reduced the CIC population and importantly reduced tumor growth and metastasis in mouse xenographs. A figure depicting the effects of PTEN on certain CICs as well as ER-signaling is present in Figure 3.

The tumor microenvironment is important in CICs. Interactions between the interleukin-8 receptor (IL-8R) and HER2 have been determined to be important in the survival of breast CICs [195]. NF-kappaB has been shown to be important in breast cancer CIC survival and HER2-dependent tumorigenesis. NF-kappaB activity can be regulated by the PI3K/PTEN/Akt/mTORC1 pathway [193].

### Targeting PI3K Pathway to Inhibit Breast Cancer Resistance to Therapy

Treatment of breast cancer patients with the aromatase inhibitor (AI) letrozole has been shown to result in suppression of the PI3K/PTEN/Akt/mTORC1 pathway [194]. This clinical study examined the expression of PI3K (p110), P-Akt, and P-mTOR by immunohistochemistry on breast cancer samples from 113 patients. The patients had been enrolled in a phase II study of letrozole or letrozole and cyclphosphamide. Either letrozole or letrozole plus cyclophosphamide-treated patients displayed a reduction in PI3K and P-mTOR expression. In contrast, expression of P-Akt did not change in the letrozole-treated patients whereas it decreased in the letrozole and cyclophosphamide-treated patients. The reduction of P-Akt expression was associated with a better response rate and reduction in Ki67 staining. The reduction in P-mTOR expression was associated with a longer disease-free survival. Thus the AI letrozole targets key components of the PI3K/PTEN/Akt/mTORC1 pathway which may be important in the successful treatment of certain breast cancers.

**Figure 4:**
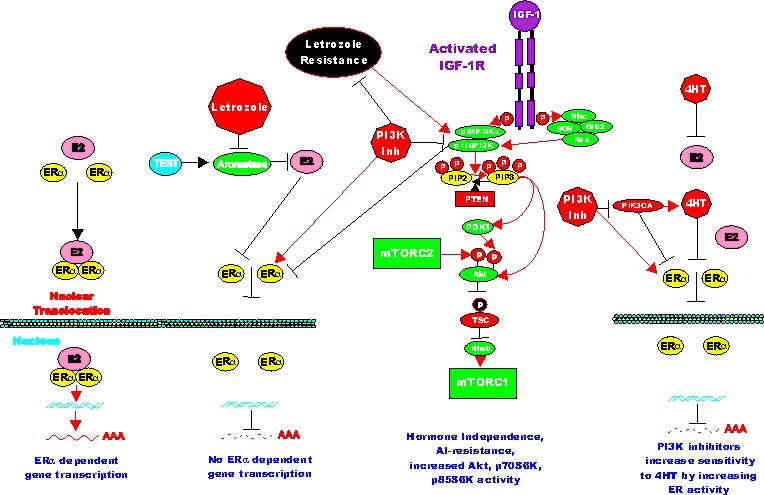
Effects of PI3K/PTEN/Akt/mTORC1 on Aromatase Resistance and Sensitivity to Tamoxifen (4HT) The aromatase inhibitor (AI) letrozole prevents the conversion of testosterone (TEST) into estrogen (ER) and hence there is no ERalpha mediated gene transcription. Letrozole alters the expression of the PI3K/PTEN/Akt/mTORC1 pathway. PI3K inhibitors will restore ERalpha mediated gene expression in Letrozole resistant cells and the cells revert to hormonal sensitivity. Likewise PI3K inhibitors will restore the sensitivity of 4HT-resistant cells to 4HT. Breast cells with mutations at PIK3CA may develop resistance to 4HT and PI3K inhibitors may restore sensitivity to 4HT.

This same group developed some letrozole-resistant MCF-7 cells by culturing the cells for prolonged periods of time in letrozole. The letrozole-resistant cells displayed elevated expression of key components of the PI3K/PTEN/Akt/mTORC1 pathway. They observed that suppression of the PI3K/PTEN/Akt/mTORC1 pathways with PI3K or mTORC1 inhibitors reversed the acquired letrozole- resistance [195]. A diagram of the effects of letrozole on ER and PI3K/PTEN/Akt/mTORC signaling and letrozole-resistance is presented in Figure 4.

### Effects of PI3K/PTEN/Akt/mTORC1 Pathway Activation and ER Expression on Breast Cancer

*PIK3CA* mutations in ER+/HER2- luminal breast cancer actually result in low levels of mTORC1 expression and these breast cancers have some of the better treatment successes after 4HT therapy [196]. These *PIK3CA* mutations are predicted to render these breast cancers sensitive to targeted therapy. The authors of this study have devised a *PIK3CA* gene expression pattern. This pattern was associated with prognosis in those breast cancers with *PIK3CA* mutations that remained ER+/HER2-, it was not associated with prognosis in breast cancers which were either HER2+ or ER-.

An inverse relationship between PI3K pathway activation and ER expression was observed in ER+ breast cancers. When the PI3K pathway was activated, lower levels of ER were detected, indicating a negative correlation. Treatment of breast cancer cells with insulin like growth factor-1 (IGF-1), which activated the PI3K pathway resulted in decreased ER expression. Likewise treatment of breast cancers with the PI3K inhibitor BEZ-235 resulted in increased ER activity as well as ER-regulated gene expression [197]. The PI3K pathway activity was increased in ER+ tumors and cell lines of the more aggressive luminal B subtype versus those of the less aggressive luminal A subtype. The PI3K inhibitor would increase the effects of 4HT on the more aggressive luminal B breast cancer, potentially by increasing ER expression and restoring sensitivity to hormone based therapies. This study suggests a potential therapeutic approach by combining PI3K inhibitors and 4HT.

The PI3K/PTEN/Akt/mTORC1 pathway is also important in the growth of breast cancers which have become hormone-independent. In four hormone-independent breast cancer cell lines created after long term estrogen deprivation, increased phosphorylation of p70S6K, p85S6K and Akt was observed [198]. Inhibition of the PI3K pathway in these hormone-independent cells resulted in apoptosis. These results indicate that some hormone-independent breast cancers may be sensitive to the combination of ER and PI3K pathway inhibitors.

Inhibition of PTEN activity has been associated with resistance to chemotherapeutic drugs, hypersensitivity to the mTORC1 inhibitor rapamycin as well as hormonal based therapies [199,200] Inhibition of PTEN expression by shRNA resulted in three ERalpha+ breast cancer cell lines that were able to grow in the absence of hormone. Furthermore the cells were resistant to 4HT and fulvestrant. Fulvestrant is an ER antagonist that functions by down regulating the ER. Knock down of PTEN also increased ERalpha transcriptional activity in MCF-7 cells, but decreased ER protein levels and transcriptional activity in T47D and MDA-361 cells. Inhibition of PTEN by shRNA increased basal and ligand-induced activation of IGF-1R and EGFR-3, documenting the effects that PTEN can have on the regulation of these upstream tyrosine kinases. Inhibition of IGF-1R or EGFR-3 restored hormonal dependency and the effects of hormonal therapy on the breast cancer cells with PTEN-knocked down. These studies indicate a possible treatment strategy for breast cancers which are either *PTEN*-negative or have decreased PTEN expression. These results document the complex interactions between hormonal- and growth factor-dependent signaling.

### Association between PIK3CA Mutations and AR Expression in Breast Cancer

An association between AR expression and *PIK3CA* mutations was observed in a study which examined AR and ER expression and *PIK3CA* mutational status in 347 breast cancer patients [201]. AR expression was higher in those breast cancers which also expressed ER and PR. In those samples that expressed AR, mutations in the *PIK3CA* kinase domain were more frequently detected than mutations in the helical domain or those lacking *PIK3CA* mutations. High AR expression was linked with an improved recurrence-free survival in 207 patients with early-stage ER+/PR+ positive tumors after hormonal-based therapy. Higher AR expression was also associated with *PIK3CA* mutations and not with *PIK3CA* WT or TNBCs. These studies indicate that AR and *PIK3CA* (as well as ER and PR) may be prognostic markers for breast cancer [201].

### Dysregulation of the PI3K/PTEN/Akt/mTORC1 Pathway in Endometrial Cancer

Endometrial cancer is one of the most frequent gynecological malignancies [202]. Many signaling pathways have been implicated in endometriod cancer [203-206]. Greater than 90% of endometriod cancers suffer from some type of mutation in the PI3K/PTEN/Akt/mTORC1 pathway. Thus, this pathway is a key therapeutic target in endometriod cancer [207,208]. Endometrioid endometrial cancers (EEC) frequently have multiple mutations at *PTEN*, *PIK3CA*, *PIK3R1* and *KRAS*. The effects of the pan-class I PI3K inhibitor GDC-0941 and the mTORC1 inhibitor temsirolimus were examined on EEC cells with concurrent *PIK3CA* and *PTEN* mutations [209]. ECC with *PIK3CA* mutations were sensitive to GDC-0941, while ECC with *PTEN* mutations were sensitive to the mTORC1 blocker. Only 2 of 6 EEC cells with *KRAS* mutations were sensitive to MEK inhibitors. The PI3K p110alpha selective inhibitor A66 was more effective than the PI3K p110beta inhibitors GSK2636771 and AZD6482 in suppressing the growth of the PTEN-mutant EEC cell lines.

### Expression of the PI3K-p110 alpha and beta Subunits in Breast Carcinomas and EEC

A study was performed on 315 invasive breast carcinomas to compare the expression of the PI3K-p110 alpha and beta subunits in these breast cancer patients. Then the expression results were correlated with clinical outcomes [210]. This immunohistochemistry study determined that overall the p110 subunits were expressed in 23.8% of invasive breast carcinomas. PI3K-p110-alpha was expressed in 11.8% and 15.2% expressed PI3K-p110-beta. This study observed that PI3K-p110-alpha expression was associated with hormone receptor expression but was not associated with overall survival. PI3K-p110-beta expression was linked to HER2 overexpression and lack of hormone receptor expression. PI3K-p110-beta+ breast cancer patients had lower age of onset, lymph node involvement and distant metastasis. Those breast cancer patients that expressed membrane PI3K-p110-beta had a worse prognosis and overall survival. These important clinical studies point to the possibility of co-targeting of HER2 and PI3K-p110-beta in certain breast cancer patients.

Recently it was determined that the *PIK3R1* (p85alpha) and *PIK3R2* (p85beta) regulatory subunits are mutated in EEC [211]. *PIK3R1* mutations were reported to occur at a higher rate in EEC than any other cancer type. Also this study demonstrated that the *PIK3R2* gene is mutated in EEC, which was previously not thought to be a cancer gene. Many *PIK3R1* and *PIK3R2* mutations are gain of function mutations. Some *PIK3R1* mutations bind and stabilize PTEN. *KRAS* mutations are also common in EECs. PI3K pathway mutations can occur in the presence of WT *PTEN* and they phenocopy *PTEN* loss as the pathway is activated.

### Deregulation of Downstream Components of the PI3K/PTEN/Akt/mTORC1 Pathway Involved in the Regulation of mRNA Translation in Breast Cancer

The PI3K/PTEN/Akt/mTORC1 pathway serves to regulate the translation of certain mRNAs which are considered difficult to translate due to their structures. The PI3K/PTEN/Akt/mTORC1 pathway can regulate the activity of key components of the translational apparatus such as eIF4E, eIF4G, 4E-BP1, rpS6, programmed cell death protein 4 (pdcd4), eEF2 and eEF2K. The expression of eIF4E, eIF4G, 4E-BP1, p4E-BP1 (T37/46), p4E-BP1 (S65), p4E-BP1 (T70), S6, pS6 (S235/236), pS6 (S240/244), pdcd4, eEF2 and eEF2K was examined in 190 hormone receptor-positive breast cancer patients [212]. This study followed the course of the breast cancer patients for 96 months. Elevated eEF2K, rpS6, and p4E-BP and decreased pdcd4 were associated with poor prognosis in hormone receptor+ breast cancer. These molecules may be prognostic markers and therapeutic targets for certain classes of breast cancer (*e.g*., hormone-responsive breast cancers).

### Involvement of GSK-3 in Breast Cancer

Phosphorylation by Akt also inhibits the activity of many key molecules involved in signaling and apoptosis. Activated Akt can stimulate carcinogenesis by inactivating proteins that normally function to limit cell growth and regulate apoptosis [213]. GSK-3beta is a down stream target of Akt. Introduction of kinase-dead GSK-3beta [GSK-3beta(KD)] into epithelial cells promoted tumorigenesis of breast and skin tumors [214]. Overexpression of constitutively-active GSK-3beta altered chemosensitivity, cell cycle arrest and tumorigenicity of breast cancers [215-218]. Inhibition of GSK-3beta, by small molecule inhibitors induced epithelial mesenchymal transition (EMT) and invasion in breast cancer [219].

The localization of GSK-3beta was altered in a study of invasive ductal carcinomas (IDC). A reduction or loss of cytoplasmic GSK-3beta in was observed in 53% of IDC examined [220]. Nuclear accumulation of GSK-3beta was detected in 35% of the IDC samples examined. This nuclear accumulation of GSK-3beta was associated positively with tumor grade [220]. A downstream target of GSK-3 is p27^Kip-1^ which is also implicated in breast cancer [221]. In addition, p70S6K can be regulated by GSK-3 and is involved in breast cancer [222]. Figure 2 depicts some of the interactions with GSK-3 and these and other signaling molecules.

GSK-3beta and beta-catenin can regulate cadherin-11 post-transcriptionally in breast and prostate cancer cells. Inactivation of GSK-3beta lead to repression of cadherin-11 mRNA and protein levels [223]. Loss of cytoplasmic GSK-3beta may promote EMT in breast cancer [223]. GSK-3 can regulate c-Myb which is important in EMT in breast cancer [224-227]. A studyl performed on breast cancer biopsies observed that inactivation of GSK-3beta was associated with elevated levels of the prolactin receptor, which is implicated in tumorigenesis [228]. The breast cancer resistance protein (BCRP) was determined to be downregulated in breast cancer cells when GSK-3beta was active, documenting that GSK-3beta can suppress active drug efflux [229]. Suppression of GSK-3beta activity by Akt phosphorylation enriched for mammary stem cells in both normal and breast cancer cells through activation of beta-catenin [230]. Thus at least with regards to mammary epithelial cells GSK-3beta activity appears to limit proliferation and suppress the stem-like cell population.

Often GSK-3beta is thought to have roles in tumor suppression. However, this is not always the case. In certain cancer types (e.g., pancreatic cancer) GSK-3beta was shown to participate in pro-inflammatory and anti-apoptotic processes by positively regulating NF-kappaB activity in the nucleus [231-233]. The roles of GSK-3beta in cancer progression remain controversial and extremely complex and may depend on the cancer type. The cellular localization of GSK-3beta is an important factor in controlling GSK-3beta ability to provide growth-limiting and survival-promoting activities. Aberrant nuclear accumulation of GSK-3beta may be important in many cancers.

The biochemical events leading to loss of functional GSK-3beta activity may also enrich for a subpopulation of CICs that demonstrate enhanced motility, clonogenicity, and drug resistance. However, the production of CICs may depend on the cancer type as well as stage of differentiation. Micro RNAs (miRs) also play important roles in CICs. TNBCs often have a high percentage of CICs [234]. Different miRs are expressed in TNBC than other breast cancer. Breast CICs often express high levels of CD44 and lower levels of CD24 (CD44↑CD24↓) than the non-CIC population which is referred frequently to as the bulk cancer. Autophagy is important in the regulation of the CIC population [235]. GSK-3 plays important roles in the regulation of autophagy and other age-related diseases [236]. Pharmacological inhibition of GSK-3 by small molecule inhibitors or deletion of GSK-3alpha leads to the prevention of autophagy.

We determined recently that GSK-3beta play important a role in MCF-7 breast cancer clonogenicity, drug resistance, and cell signaling [218]. Introduction of GSK-3beta(KD) into MCF-7 cells resulted in increases in both anchorage-dependent and anchorage-independent clonogenicity compared to cells transfected with the GSK-3beta(WT) construct. This increase in clonogenicity was observed when doxorubicin was absent or present. More colonies were observed in MCF-7/GSK-3beta(KD) cells than MCF-7/GSK-3beta(WT) or MCF-7/GSK-3beta(A9) cells when the cells were treated with doxorubicin. MCF-7/GSK-3beta(A9) cells with constitutive GSK-3beta kinase activity displayed higher anchorage-dependent, but not anchorage-independent, clonogenicity than MCF-7/GSK-3beta(WT) cells. Anchorage-independent growth is one measurement of transformed cells. In essence, it measures the ability of the cells to grow without adherence to a tissue culture plate surface. Cells which are more transformed will form more colonies in soft agar than cells which are either “not”-tranformed or “less”-transformed. GSK-3beta may play multiple roles by both limiting proliferation under certain conditions and allowing cell growth in others. In some cases GSK-3beta functions as a tumor promoter by phosphorylation of Axin which leads to beta-catenin stabilization and potentially cancer. In contrast, other cases GSK-3beta acts as a tumor suppressor and induces beta-catenin phosphorylation and proteasomal degradation.

When the cells were plated n doxorubicin, the anchorage-independent colony forming ability of MCF-7/GSK-3beta(KD) cells in doxorubicin was enhanced five-fold in comparison to MCF-7/GSK-3beta(WT) cells. Our results indicated that GSK-3beta activity influences breast cancer proliferation, motility and response to chemotherapy. Loss of GSK-3beta kinase activity may confer survival advantages by upregulating factors involved in cell cycle progression, prevention of apoptosis and anchorage-independence.

Resistance to doxorubicin and 4HT was increased in MCF-7/GSK-3beta(KD) cells in comparison to MCF-7/GSK-3beta(WT) cells. However, drug resistant MCF-7/GSK-3beta(KD) cells responded to mTOR inhibition by treatment with rapamycin. Additionally, a combination treatment of consisting of a MEK inhibitor and doxorubicin or 4HT was determined to have a synergistic effect that eliminated drug resistance in MCF-7/GSK-3beta(KD) cells. Targeting signaling molecules involved with the PI3K/PTEN/Akt/mTORC1 and Raf/MEK/ERK pathways may be appropriate to overcome resistance to chemo- and hormonal therapy. Genetic alterations which result in abnormal GSK-3beta activity should be taken into considered when designing a course of breast cancer therapy. Combination drug/inhibitor treatments could be advantageous by lowering the concentration of the chemotherapeutic drug and reduce therapy-related side effects.

There are alternate routes of GSK-3beta activation that are independent of Akt. To add to the complexity, GSK-3beta plays roles in the Wnt/beta-catenin pathway by being a component of the beta-catenin destruction complex as well as in the phosphorylation of Axin which results in the stabilization of Axin. This interaction of GSK-3 with the Axin protein complex prevents Akt from accessing and phosphorylating GSK-3beta. There are likely different cellular pools of GSK-3beta that are under separate regulation by Wnt and Akt, in which the activation of one does not affect the other. Simultaneous Akt and GSK-3beta activity has been observed in pancreatic and colon cancer cell lines, indicating that increased Akt expression does not always result with decreased GSK-3beta activity [231]. Since certain pools of GSK-3beta may remain active in cancer, the precise cellular localization of these pools may also be an important factor involved in their regulation. GSK-3beta is thought to be active predominantly in the cytoplasm, but it can also translocate to the nucleus. The nuclear activities of GSK-3beta may appear to conflict with its cytosolic roles. Aberrant nuclear accumulation of GSK-3beta has been observed in certain cancers [231]. A nuclear localization signal (NLS) is present in the basic domain of GSK-3beta [237]. Increased translocation of GSK-3beta to the nucleus may be involved with cancer progression.

### Involvement of the Wnt Signaling Pathway in ERalpha+ Breast Cancers with PIK3CA Mutations

The *PIK3CA* gene is mutated in 30-40% of ERalpha+ breast cancers. Gene expression profiling of 249 ER-alpha+ breast tumors revealed that nineteen genes were differently expressed in *PIK3CA*-mutated tumors in comparison to samples lacking *PIK3CA* mutations. Interesting *PIK3CA* mutations were associated with over-expression of several genes critical for the Wnt signaling pathway [*WNT^5^A*, transcription factor 7-like 2 (T-cell specific, HMG-box (*TCF^7^L^2^*), msh homeobox 2 (*MSX^2^*), tumor necrosis factor receptor superfamily, member 11b (*TNFRSF^11^B*)], as well as genes important in the regulation of gene transcription [SEC^14^-like 2 (*SEC^14^L^2^*), transcription factor AP-2 beta (activating enhancer binding protein 2 beta) (*TFAP^2^B*), nuclear receptor interacting protein 3 (*NRIP^3^*)] and metal ion binding [cytochrome P450, family 4, subfamily Z, polypeptide 1 (*CYP4Z1*), cytochrome P450, family 4, subfamily Z, polypeptide 2, pseudogene (*CYP4Z2P*), solute carrier family 40 (iron-regulated transporter), member 1 (*SLC40A1)*, lactotransferrin (*LTF*), LIM and calponin homology domains 1 (*LIMCH1*) [238]. These studies suggest that targeting the Wnt signaling pathway may be an appropriate approach to treat certain ERalpha+ breast cancers containing *PIK3CA* mutations.

### Interactions between PI3K/PTEN/Akt/mTORC1 and Raf/MEK/ERK Pathways in Breast Cancer and Drug Resistance

**Figure 5:**
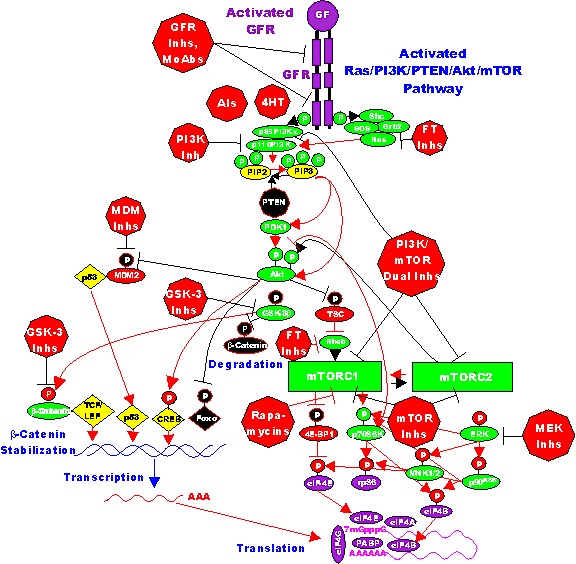
Sites of Targeting the EGFR/PI3K/PTEN/Akt/mTORC Pathway with Small Molecule Membrane-Permeable Inhibitors and Monoclonal Antibodies (MoAbs) The PI3K/PTEN/Akt/mTORC1 pathway is regulated by Ras (indicated in green ovals), as well as various upstream growth factor receptors (indicated in purple) and PTEN indicated in a black rectangle. Sites where various small molecule inhibitors suppress this pathway are indicated by red octagons. The downstream transcription factors regulated by this pathway are indicated in yellow diamond shaped outlines. The Ras/Raf/MEK/ERK pathway also interacts with key proteins involved in protein translation (indicated in green ovals). The two pathways aid in the assembly of the protein translation complex (indicated in purple ovals) responsible for the translation of “weak” mRNAs (indicated in a red line folding over on itself) important in the prevention of apoptosis. Other key proteins inactivated by PI3K/PTEN/Akt/mTORC1 pathway (e.g., TSC, PTEN, beta-catenin, Foxo and 4E-BP1 are indicated by black ovals, diamonds and rectangles. Red arrows indicate activating events in pathways. Blocked black arrows indicating inactivating events in pathways. GF = growth factor, GFR = growth factor receptor.

The Raf/MEK/ERK pathway is also important in breast cancer drug resistance [239,240]. The Raf/MEK/ERK pathway often interacts with the PI3K/PTEN/Akt/mTORC1 pathway and the two pathways often co regulate many signaling molecules such as p70S6K, eIF-4B, eIF-4E, rpS6 and others. A figure illustrating some of the sites of targeting these pathways is present in Figure 5. A recent component which has been shown to be involved in breast cancer drug resistance is the phosphatase DUSP4 which removes the phosphate from active ERK1,2. Decreased expression of DUSP4 is associated with breast cancer drug resistance and increased expression of active ERK1,2 [241].

Other studies have indicated that increased MAPK signaling is a predicator of resistance to successful endocrine therapy of breast cancer patients [242]. This study included 114 women who were ER-alpha+ that were randomly assigned to neoadjuvant letrozole or letrozole plus metronomic cyclophosphamide (frequent low doses of cyclophosphamide). The expression of twenty-four proteins involved in apoptosis, cell survival, hypoxia, angiogenesis and signaling were examined by immunohistochemistry in pretreatment samples. The ages, sizes of the tumors, the nodal status of the tumor, tumor grade, histological type and treatment were also followed. 81% of the patients had a disease response, 43% achieved a complete clinical response and 19% did not respond. This study determined that increased phosphorylated ER-alpha and decreased phosphorylated ERK1,2 were factors associated with complete response to therapy. Phosphorylated and activated ER-alpha was an independent factor for sensitivity to chemoendocrine therapy. In contrast, HIF-alpha and phosphorylated ERK1/2 were independent factors associated with resistance to therapy. These and other results indicating that targeting ERK (*e.g.,* with MEK inhibitors) may be effective in reducing the drug resistance of certain breast cancers.

### Interactions Between Raf-1 and Aurora Kinase Signaling in ER+ Breast Cancer

Recently, it has been shown that there is a Raf-1-mediated involvement of Aurora-A kinase signaling which is important in regulating the balance between EMT and MET in ER+ breast cancer cells. This interaction is also important in chemoresistance and regulating the expression of many genes involved in EMT and MET. The interactions between Raf-1 and Aurora-1 kinase signaling altered the metastatic potential of the cells. Constitutive activation of Raf-1 oncogenic signaling induced HER-2 overexpression and resulted in the development of distant metastases in ERα+ MCF-7/ΔRaf-1 breast cancer xenografts. These distant metastases in xenograft models were associated with activation of MET as characterized by reduced expression of EMT-inducer genes (*TGFB2*, *TWIST1* and *FOXC1*) while overexpression of the *BMB7*, *CXCR7* and *EGR* genes. Constitutive activation of Raf/MEK/MAPK oncogenic signaling during tumor growth promoted the development of metastatic lesions from primary tumors by activating MET [174].

Further studies by this same group demonstrated that constitutive activation of Raf-1 oncogenic signaling induced stabilization and accumulation of Aurora-A mitotic kinase. This promoted EMT and stemness in ER+ MCF-7/ΔRaf-1 cells. The EMT transition was associated by reduced expression of CD24 and ERalpha, while HER2 and CD44 were upregulated. The upregulation of the stemness gene SOX2 was linked to acquisition of multiple stem cell-like properties. Namely the cells displayed an enhanced ability to form mammospheres *in vitro* and self-renewal *in vivo*. The aberrant Aurora-A kinase activity induced phosphorylation and nuclear translocation of SMAD5, indicating a novel interplay between Aurora-A and SMAD5 signaling pathways in the development of EMT, stemness and ultimately tumor progression. Pharmacological and molecular inhibition of Aurora-A kinase activity restored the CD24↑ epithelial phenotype that was coupled with ERalpha expression, downregulation of HER2, inhibition of EMT and impaired self-renewal ability and suppression of distant metastases. These findings demonstrated the importance of Aurora-A kinase in the activation of EMT pathway responsible for the development of distant metastases in ERalpha+ breast cancer cells. This study has translational implications because it highlighted the mitotic kinase Aurora-A as a novel, promising therapeutic target to eliminate invasive breast cancer cells and improve the disease-free and overall survival of ERalpha+ breast cancer patients resistant to conventional endocrine therapy [164].

Further studies indicated that cross-talk between ERalpha+ and Raf/MEK/ERK signaling pathways is a key oncogenic axis which is responsible in part for the development of ER-independent growth of breast cancers which were initially ERalpha+ and hormone sensitive. In a metastatic breast cancer xenograft model harboring constitutive activation of Raf-1, the link between aberrant Raf/MEK/ERK signaling and development of endocrine resistance through abrogation of the ERα signaling axis was elucidated. The Aurora-A mitotic kinase was demonstrated to be important in the development of endocrine resistance. Activation of SMAD5 nuclear signaling was detected as well as down-regulation of ERalpha. These results suggest that the development of novel molecular therapies targeting the Aurora-A/SMAD5 oncogenic axis may be beneficial for the selective eradication of endocrine resistant ERalpha- cancer cells from the bulk tumor with benefits for breast cancer patients [243].

### Relationship between Raf-1/p53/Aurora Kinase and Centrosome Amplification in Breast Cancer

Centrosome amplification has been shown to play key roles in the origin of chromosomal instability (CIN) that affects cancer development and progression. The relationships between induction of genotoxic stress, activation of cyclin-A/Cdk2 and Aurora-A and development of centrosome amplification were investigated in MCF-7 breast cancer cell lines harboring a dominant negative (DN) p53 mutation (vMCF-7DNp53). Genotoxic stress was induced in the MCF-7 cells harboring the DN p53 by treatment with hydroxyurea (HU) which induced centrosome amplification. Aurora-A kinase activity was linked with centrosome amplication. The Aurora-A kinase-induced centrosome amplification was determined to be mediated by Cdk2 kinase. Molecular inhibition of Cdk2 activity by SU9516 suppressed Aurora-A centrosomal localization and consequent centrosome amplification. MCF-7 cells harboring constitutively-activated Raf-1 displayed high levels of endogenous cyclin-A. Targeting of Aurora-A by the Aurora kinase inhibitor Alisertib reduced cyclin-A expression. Thus there is a positive feed-back loop between cyclin-A/Cdk2 and Aurora-A pathways in the development of centrosome amplification in breast cancer cells which may be which may provide additional approaches to target drug resistant breast cancer patients [244]. These studies document the key interactions between the Raf/MEK/ERK and Aurora kinase pathways which may be important in regulating breast cancer progression and serve as targets for therapeutic intervention.

### Breast Cancer Therapy

The most commonly utilized treatment for breast cancer is surgical resection with adjuvant chemotherapy, hormone therapy or radiation (National Cancer Institute, 2013). Radiation and chemotherapy are effective in killing or limiting the growth of actively dividing cancer cells through various mechanisms including the production of oxygen free radicals, DNA damage, and subsequent apoptosis [245-251]. Common chemotherapeutic drugs used to treat breast cancer include the anthracyclines, taxanes, 5-fluorouracil, cyclophosphamide, and methotrexate [252]. Doxorubicin, also known as adriamycin, is in the anthracycline class of antibiotic chemotherapeutic drugs. These compounds work by intercalating between adjacent DNA base pairs and inhibiting topoisomerase II, thereby interfering with DNA, RNA, and protein synthesis.

### The effects of combining chemotherapeutic drugs have been analyzed by clinical trials

A phase III, clinical trial with 1491 patients with node-positive, early breast cancer were randomly assigned to adjuvant treatment with docetaxel, doxorubicin, and cyclophosphamide (ACT) or fluorouracil, doxorubicin, and cyclophosphamide (FAC) every 3 weeks for six cycles. The 10 year follow-up analysis of disease-free survival, overall survival, and long-term safety in the clinical trial was reported [253]. In general, breast cancer patients in ACT treatment group had better disease-free survival relative to breast cancer patients in the FAC treatment group. In this study, hormone receptor, and HER2 status and nodal involvement status did not appear to change the disease-free survival between the enhanced survival in ACT treatment group vs. the FAC treatment group. This study also documented that there were a significant percentage of patients who had a decrease in ventricular ejections secondary to anthracycline therapy.

An additional study evaluated the effects of ACT and FAC treatments on node negative breast cancer patients over a 5 year observation period. This study was performed as it was known previously that ACT is superior to FAC when used as adjuvant therapy in women with node-positive breast cancer but the effects of taxanes on breast cancer patients with node cancer were not known [254]. The breast cancer patients in the two treatment groups were examined for 77 months. The disease-free survival was higher in the ACT group (87.8%) than the FAC group (81.8%). Thus the ACT treatment may be appropriate for some high risk, lymph node negative breast cancer patients.

### Hormonal Therapy Combined with mTORC1 Blockage

Another common treatment option for breast cancer consists of a hormonal-based approach [255]. Many early stage breast cancers overexpress various isoforms of the ER, making their growth dependent on estrogen. 4HT is a selective ER modulator. 4HT can block estrogen signaling by competitively binding the ER and antagonizing its proliferative effects [256]. Once a breast cancer has undergone additional molecular changes allowing it to overcome estrogen-dependence, hormonal therapy is no longer effective, however, certain inhibitors can reverse the estrogen-independence (see Figures 3 and 4) [257-261].

Exemestane is an AI and prevents conversion of testosterone into estrogen. The quality of life was observed to be better in hormone-responsive metastatic breast cancer patients treated with exemestane and the mTORC1 blocker everolimus than patients treated with exemestane by itself. In the BOLERO-3 study with herceptin-resistant metastatic breast cancer patients, treatment with everolimus, herceptin and vinorelbine was more effective than treatment of with herceptin and vinorelbine [262]. Vinorelbine (Navelbine) is anti-mitotic drug [261]. It is used to treat non-small cell lung cancer and metastatic breast cancer. Thus suppressing mTORC1 activity could enhance the effectiveness of exemestane and Herceptin in different types of breast cancer patients.

Previously it was thought that hormonal therapy would only be effective in treatment of breast cancer cells which express ER-alpha. Triple negative breast and HER2+ cancers often do not expresses ER-alpha, but some express ER-beta and may be sensitive to hormonal therapy [262]. The expression of hormonal receptors (ER, PR) and HER2 may change with the progression of breast cancer into metastatic cancer. In addition, the combination of hormonal based therapy can be enhanced by the addition of herceptin and mTORC1 blockers in some cases.

### Antibody Therapy of Breast Cancer

**Figure 6:**
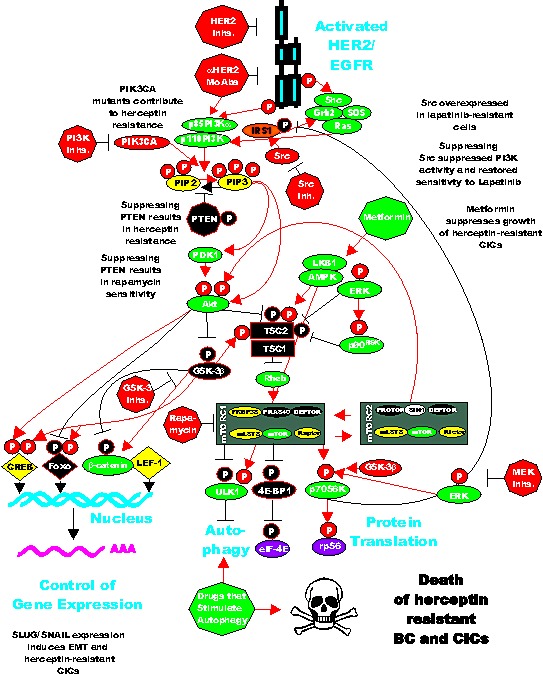
Sites of Resistance in the HER2/PI3K/PTEN/Akt/mTORC Pathway and Potential Sites for Intervention with Small Molecule Membrane-Permeable Inhibitors and Monoclonal Antibodies (MoAbs) The HER2/EGFR receptor is indicated in a blue figure. The downstream PI3K/PTEN/Akt/mTORC1 pathway is regulated by Ras (indicated in green ovals), PTEN indicated in a black octagon, activated Src is indicated by a red oval, IRS1 is indicated by an orange oval. Sites where various small molecule inhibitors suppress this pathway are indicated by red octagons. Sites which stimulate proteins involved in autophagy are indicated by green octagons and ovals. The Serine/threonine-protein kinase ULK1 (ULK1) which is regulated by mTORC1 is indicated in a black oval. The downstream transcription factors regulated by this pathway are indicated in yellow diamond shaped outlines. The Ras/Raf/MEK/ERK pathway also interacts with key proteins involved in protein translation (indicated in green ovals). The two pathways aid in the assembly of the protein translation complex (indicated in purple ovals) responsible for the translation of “weak” mRNAs (indicated in a red line folding over on itself) important in the prevention of apoptosis. Other key proteins inactivated by PI3K/PTEN/Akt/mTORC1 pathway (e.g., TSC, PTEN, GSK-3beta, beta-catenin, Foxo and 4E-BP1 are indicated by black ovals, diamonds and rectangles. Red arrows indicate activating events in pathways. Blocked black arrows indicating inactivating events in pathways.

The genetically engineered antibody herceptin (trastuzumab) is used to treat HER2+ breast cancers. Combining herceptin with chemotherapy increases survival and response rates [263]. However a significant problem with chemotherapy is cardiotoxicity. Herceptin therapy is also expensive and not always covered by insurance companies [264]. A diagram depicting the targeting of HER2 and other signaling molecules and sites where herceptin-resistance occurs is presented in Figure 6.

Pertuzumab (Perjeta(®) is a newer antibody developed by Genentech/Roche. Pertuzumab and herceptin binding to different sites on HER2 and the combined administration of pertuzumab and herceptin had synergistic inhibitory effects on HER2+ breast cancers [265].

Pertuzumab prevents the dimerization of HER2 with other EGFR family members (EGFR1, EGFR3, EGFR4) [266]. Pertuzumab prevents the interaction of HER2 with EGFR3 and subsequent activation of the PI3K/PTEN/Akt/mTOR pathway. This has been proposed to be in part responsible to the anti-cancer effects of pertuzumab [267].

Initial studies with just pertuzumab did not reveal impressive results in suppressing cancer growth. However, in the more recent phase III CLEOPATA trial have revealed that combination of pertuzumab with herceptin and docetaxel were promising and significantly improved prolonged progression-free survival for a first-line treatment of HER2+ metastatic breast cancer and importantly no increase in cardio-toxic effects were observed [268]. Pertuzumab received approval by the FDA to treat HER2+ metastatic breast cancer patients in 2012. Pertuzumab in combination with herceptin and a taxol has been approved by the FDA and is being evaluated as a first line treatment option for HER2+ metastatic breast cancer patients who have not been previously treated with anti-HER2 therapy or chemotherapy [269].

Pertuzumab is being further evaluated in the following clinical trials: MARIANNE, for advanced breast cancer, NEOSPHERE, for early breast cancer, TRYPAHENA, for HER2+ stage II/III breast cancer and APHINITY for HER2+ nonmetastatic breast cancer [270].

Trastuzumab emtansine is an antibody-drug modification of herceptin which is now linked with the cytotoxic agent mertansine (DM1). It is abbreviated T-DM1 (trastuzumab-DM1) and also called Kadcyla, ado-trastuzumab emtansine and PRO132365. Mertansine inhibits cell growth by binding to tubulin [271]. Herceptin targets the antibody-drug conjugate to HER+ cells [272]. T-DM1 is more effective than Herceptin in the treatment of HER+ advanced metastatic breast cancer patients. It was evaluated in the EMILIA phase III clinical trial. This trial consisted of 991 cancer patients with unrectable, locally advanced or metastatic HER+ breast cancer which had been previously treated with herceptin and taxanes with breast cancer patients treated with capecitabine (Xeloda) plus lapatinib (Tykerb). Progression free survival was enhanced in breast cancer patients treated with T-DM1. It has been approved by the FDA for the treatment of HER2+ advanced metastatic breast cancer patients. T-DM1 has also been evaluated in other clinical trials such as MARIANNE, which compares the effectiveness of the taxanes (docetaxel or paclitaxel) combined with Herceptin vs T-DM1 by itself vs T-DM1 plus pertuzumab as a first line therapy for HER2+ breast cancers. Patients in this study are HER2+, which have either unresectable locally advanced or metastatic breast cancer. T-DM1 is also being investigated in the TH3RESA clinical trial which is evaluating the effectiveness T-DM1 in HER2+ metastatic breast cancer patients that were previously treated with Herceptin and the EGFR2 kinase inhibitor lapatinib. T-DM1 is also being evaluated in gastric cancers in clinical trials. Tables 1-3 and Supplementary Tables 1-3 list the various clinical trials in breast cancer which are listed on the ClinicalTrial.gov website.

**Table 1:** Clinical Trials with Single Targeted Agent Treatments (Monotherapy)

Official Trial Name	Clinical Trial #	Phase of Trial	Type of Cancer Patient in Trial	Status of Trial	Intervention	Publications
Phase II Trial of Akt Inhibitor MK2206 in Patients With Advanced Breast Cancer Who Have Tumors With a PIK3CA Mutation, or an AKT Mutation, and/or PTEN Loss/PTEN Mutation	NCT01277757	II	Male Breast Cancer (BC), recurrent BC, stage IIIB BC, stage IIIC BC, stage IV BC	Recruiting	MK2206 Akt inhibitor	Not Provided
Pre-surgical Evaluation of MK-2206 in Patients With Operable Invasive Breast Cancer	NCT01319539	II	ER- BC, ER+ BC, HER2- BC, HER2+ BC, PR- BC, PR+ BC, stage IB BC, stage II BC, stage IIIA BC, stage IIIB BC, stage IIIC BC, TNBC	Ongoing, but not recruiting participants	MK2206	Not provided
A Phase I, Open-Label, Two-Stage Study to Investigate the Safety, Tolerability, Pharmacokinetics and Pharmacodynamics of the Oral AKT Inhibitor GSK2141795 in Subjects With Solid Tumors or Lymphomas	NCT00920257	I	Cancer	Completed	GSK2141795 Akt Inhibitor	Not provided
A Phase II Trial of BKM120 (a PI3K Inhibitor) in Patients With Triple Negative Metastatic Breast Cancer	NCT01629615	II	BC	Currently recruiting participants	BKM120 PI3K Inhibitor	Not provided
A Phase I/II, Multi-center, Open-label Study of BEZ235, Administered Orally on a Continuous Daily Dosing Schedule in Adult Patients With Advanced Solid Malignancies Including Patients With Advanced Breast Cancer	NCT00620594	I	BC, Advanced Solid Tumors, Cowden Syndrome	Completed	BEZ235 PI3K/mTOR Inhibitor	Not provided
A Phase II Trial of Short-Term Everolimus (RAD001) to Predict Response in Women With Operable Breast Cancer	NCT00855114	II	BC	Withdrawn	Everolimus (mTORC1 blocker)	Not provided
Phase II Trial of CCI-779 (Temsirolimus) in Patients With Locally Advanced or Metastatic Breast Cancer	NCT00376688	II	Male BC, Recurrent BC, Stage IIIA, Stage IIIB, Stage IIIC, Stage IV BC	Ongoing but not recruiting	Temsirolimus (mTORC1 blocker)	Not provided
A Phase Ib/II Study Investigating the Combination of Everolimus With Trastuzumab and Paclitaxel in Patients With HER2-overexpressing Metastatic Breast Cancer	NCT00426556	I	Metastatic BC	Completed	Everolimus	Not provided
A Phase II Trial of RAD001 in Triple Negative Metastatic Breast Cancer	NCT00827567	II	Breast cancer	Terminated	RAD 001 (everolimus)	[337-339]
A Phase Ib Study Investigating the Combination of Everolimus With Trastuzumab and Vinorelbine in Patients With HER2-overexpressing Metastatic Breast Cancer	NCT00426530	I	Breast Neoplasms, Neoplasm Metastasis	Completed	Everolimus	[340]
Neoadjuvant Phase II Study Of Everolimus Plus Cisplatin In Triple Negative Breast Cancer Patients With Residual Disease After Standard Chemotherapy	NCT01931163	II	BC, TNBC	Recruiting	Everolimus	Not provided
Randomized, Double Blind, Multicentric Phase III Trial Evaluating the Safety and Benefit of Adding Everolimus to Adjuvant Hormone Therapy in Women With Poor Prognosis, ER+ and HER2- Primary Breast Cancer Who Remain Free of Disease After Receiving 3 Years of Adjuvant Hormone Therapy	NCT01805271	III	ER+, HER2- BC	Recruiting	Everolimus	Not provided
RADAR: A Randomized Discontinuation Phase II Study to Determine the Efficacy of RAD001 in Breast Cancer Patients With Bone Metastases	NCT00466102	II	BC	Ongoing, but not recruiting	RAD001	Not provided
Phase II Trial of RAD001 Plus Carboplatin in Patients With Triple-Negative Metastatic Breast Cancer	NCT01127763	II	BC	Ongoing, but not recruiting	RAD001	Not provided
A Randomized Phase II Study of Two Different Schedules of RAD001C in Patients With Recurrent/Metastatic Breast Cancer	NCT00255788	II	BC	Completed	Everolimus	Not provided
A Randomized Study of mTOR Inhibition by RAD001 (Everolimus) in Invasive Breast Cancer Patients After Pre-operative Use of Anthracycline and/or Taxane-based Chemotherapy	NCT01088893	II	BC	Unknown because information has not been verified recently	Everolimus	Not provided
A Multicenter Randomized, Double Blind, Placebo- Controlled, Phase II Study to Compare Endocrine Treatment Alone Versus Endocrine Treatment With Everolimus in Patients With HR+/HER2- Metastatic Breast Cancer and Progression After Previous Treatment With Exemestane and Everolimus	NCT01773460	III	Metastatic BC	Recruiting	Everolimus	Not provided
Influence of Exceptional Patient Characteristics on Everolimus Exposure	NCT01948960	IV	BC	Recruiting	Everolimus	Not provided
A Phase Ib Study Administering Rapamycin (Sirolimus) With Grapefruit Juice in Patients With Advanced Malignancies	NCT00375245	I	Tumors, Neoplasm Metastasis	Completed	Rapamycin (sirolimus) Other: Grapefruit Juice	Not provided
A Phase Ib, Open-label Study to Evaluate RAD001 as Monotherapy Treatment in Chinese Patients With Advanced Pulmonary Neuroendocrine Tumor	NCT01175096	I/II	Neuroendocrine Tumors, Carcinoid Tumor	Recruitment unknown because information has not been verified recently	RAD001 (everolimus, Afinitor®)	Not provided
A Phase 1/2, Multi-Center, Open-Label, Dose Finding Study to Assess the Safety, Tolerability, Pharmacokinetics and Preliminary Efficacy of the mTOR Kinase Inhibitor CC-223 Administered Orally to Subjects With Advanced Solid Tumors, Non-Hodgkin Lymphoma or Multiple Myeloma.	NCT01177397	I/II	Multiple Myeloma, Diffuse Large B-Cell Lymphoma, Glioblastoma Multiforme, Hepatocellular Carcinoma, Non-Small Cell Lung Cancer, Neuroendocrine Tumors of Non-Pancreatic Origin, Hormone Receptor-Positive BC	Currently recruiting participants	CC-223	Not provided
A Randomized Study of mTOR Inhibition by RAD001 (Everolimus) in Invasive Breast Cancer Patients After Pre-operative Use of Anthracycline and/or Taxane-based Chemotherapy	NCT01088893	II	BC	Recruitment unknown because the information has not been verified recently	Everolimus	Not provided

**Table 2:** Combined Targeted Agent Treatments

Official Trial Name	Clinical Trial #	Phase of Trial	Type of Cancer Patient in Trial	Status of Trial	Intervention	Publications
An Open-Label, Two Part, Phase I/II Study to Investigate the Safety, Pharmacokinetics, Pharmacodynamics, and Clinical Activity of the MEK Inhibitor GSK1120212 in Combination With the AKT Inhibitor GSK2110183 in Subjects With Solid Tumors and Multiple Myeloma	NCT01476137	I	Cancer	Completed	GSK1120212, GSK2110183	Not provided
A Phase Ib, Open-label, Multi-center, Dose-escalation and Expansion Study of an Orally Administered Combination of BKM120 Plus MEK162 in Adult Patients With Selected Advanced Solid Tumors	NCT01363232	I	Advanced Solid Tumors, Selected Solid Tumors	Ongoing, but not participating	BKM120 (a pan-class I PI3K inhibitor)+ MEK162 (MEK inhibitor)	Not provided
A Phase Ib, Open-label, Multi-center, Dose-escalation Study of Oral BKM120 in Combination With Oral GSK1120212 in Adult Patients With Selected Advanced Solid Tumors.	NCT01155453	I	Advanced Solid Tumors, Selected Solid Tumors	Ongoing, but not recruiting	BKM120, GSK1120212	Not provided
A Phase I/II Trial of an Oral MTOR Protein Kinase Inhibitor (Everolimus, RAD001) in Combination With an Oral EGFR Tyrosine Kinase Inhibitor (Erlotinib, Tarceva™) In Patients With Metastatic Breast Cancer	NCT00574366	I	BC	Completed	Erlotinib (EGFR1 inhibitor), Everolimus (RAD001)	Not provided
An Open-label, Multi-center Phase I Dose-finding Study of RAD001 (Everolimus, Afinitor®) in Combination With BEZ235 in Patients With Advanced Solid Tumors	NCT01482156	I	Advanced Solid Tumors, Metastatic BC, Metastatic Renal Cell Carcinoma	Ongoing, but not recruiting	RAD001 + BEZ235, a dual inhibitor of PI3K and mTOR	Not provided
Phase I/II Trial of IMC-A12 in Combination With Temsirolimus in Patients With Metastatic Breast Cancer	NCT00699491	I/II	Male BC, Recurrent BC, Stage IV BC	Ongoing, but not recruiting	Cixutumumab (an IGF-1R inhibitor), temsirolimus	Not provided
Phase I Clinical Trial of Temsirolimus and Vinorelbine in Advanced Solid Tumors.	NCT01155258	I	Extensive Stage Small Cell Lung Cancer, Hereditary Paraganglioma, Male BC, Malignant Paraganglioma, Metastatic Gastrointestinal Carcinoid Tumor, Metastatic Pheochromocytoma, Pancreatic Polypeptide Tumor, Recurrent Breast Cancer, Recurrent Cervical Cancer, Recurrent Endometrial Carcinoma, Recurrent Gastrointestinal Carcinoid Tumor, Recurrent Islet Cell Carcinoma Recurrent Neuroendocrine Carcinoma of the Skin, Recurrent Non-small Cell Lung Cancer, Recurrent Ovarian Epithelial Cancer Recurrent Ovarian Germ Cell Tumor, Recurrent Pheochromocytoma, -Recurrent Prostate Cancer, Recurrent Renal Cell Cancer, Recurrent Small Cell Lung Cancer, Recurrent Uterine Sarcoma, Regional Gastrointestinal Carcinoid Tumor, Regional Pheochromocytoma, Stage III Cervical Cancer, Stage III Endometrial Carcinoma, Stage III Neuroendocrine Carcinoma of the Skin, Stage III Ovarian Epithelial Cancer, Stage III Ovarian Germ Cell Tumor, Stage III Prostate Cancer, Stage III Renal Cell Cancer, Stage III Uterine Sarcoma, Stage IIIA BC, Stage IIIA Non-small Cell Lung Cancer, Stage IIIB BC, Stage IIIB Non-small Cell Lung Cancer, Stage IIIC BC, Stage IV BC, Stage IV Endometrial Carcinoma, Stage IV Neuroendocrine Carcinoma of the Skin, Stage IV Non-small Cell Lung Cancer, Stage IV Ovarian Epithelial Cancer, Stage IV Ovarian Germ Cell Tumor, Stage IV Prostate Cancer, Stage IV Renal Cell Cancer, Stage IV Uterine Sarcoma, Stage IVA Cervical Cancer, Stage IVB Cervical Cancer Thyroid Gland Medullary Carcinoma	Ongoing, but not recruiting	Temsirolimus, vinorelbine ditartrate (an anti-mitotic vinca alkaloid)	Not provided
A Phase I Study of Temsirolimus in Combination With Metformin in Advanced Solid Tumours	NCT00659568	I	BC, Endometrial Cancer, Kidney Cancer Lung Cancer, Lymphoma, Unspecified Adult Solid Tumor, Protocol Specific	Completed	Metformin hydrochloride, temsirolimus	Not provided
An Open-Label, Phase Ib Dose Escalation Trial of Oral Combination Therapy With MSC1936369B and SAR245409 in Subjects With Locally Advanced or Metastatic Solid Tumors	NCT01390818	I	Locally Advanced Solid Tumor, Metastatic Solid Tumor, BC, Non Small Cell Lung Cancer, Melanoma, Colorectal Cancer	Ongoing, but not recruiting	MSC1936369B (a MEK inhibitor) and SAR245409 (a dual PI3K/mTOR inhibitor)	Not provided
A Phase I Study of BKM120 and Everolimus in Advanced Solid Malignancies	NCT01470209	I	Solid Tumors	Recruiting participants	BKM120, everolimus	Not provided
A Phase Ib, Open-label, Multi-center, Dose-escalation and Expansion Study of an Orally Administered Combination of BEZ235 Plus MEK162 in Adult Patients With Selected Advanced Solid Tumors	NCT01337765	I	Unspecified Adult Solid Tumor, Protocol Specific Solid Tumor	Completed	BEZ235, MEK162	Not provided
A Clinical Trial to Qualify the Growth Factor Signature (GFS) as an Intermediate Biomarker of Response for Development of PI3K-Pathway Inhibitors in Patients With Breast Cancer	NCT01220570	I	BC	Completed	Ridaforolimus (mTORC1 blocker), Dalotuzumab (IGF-1R inhibitor)	Not provided
Phase I Parallel Protocol of MK-8669 (Ridaforolimus) + MK-2206 and MK-8669 (Ridaforolimus) + MK-0752 Doublets (MK-MK) in Patients With Advanced Cancer	NCT01295632	I	Advanced Cancer	Ongoing, but not recruiting	Ridaforolimus, MK-0752 (gamma secretase inhibitor, a Notch signaling pathway inhibitor). MK-2206	Not provided

**Table 3:** Targeted Agents in Combination with Cytotoxic Therapy

Official Trial Name	Clinical Trial #	Phase of Trial	Type of Cancer Patient in Trial	Status of Trial	Intervention	Publications
Phase Ib Dose Escalation and Biomarker Study of MK-2206 in Combination With Standard Doses of Weekly Paclitaxel in Patients With Locally Advanced or Metastatic Solid Tumors With an Expansion in Advanced Breast Cancer	NCT01263145	I	Recurrent BC, -Stage IV BC, Unspecified Adult Solid Tumor	Active, not recruiting	MK2206, paclitaxel	Not Provided
A Phase I-II Study of Triciribine Phosphate Monohydrate (TCN-PM) Plus Sequential Weekly Paclitaxel Followed by Dose-Dense Doxorubicin and Cyclophosphamide in Patients With Metastatic and Locally Advanced Breast Cancer	NCT01697293	I/ II	Metastatic BC, Carcinoma Breast Stage IV	Currently recruiting participants	Triciribine (an Akt inhibitor), paclitaxel, doxorubicin, cyclophosphamide	Not provided
A Phase Ib/II Trial of GDC-0941 (a PI3K Inhibitor) in Combination With Cisplatin in Patients With Androgen Receptor Negative Triple Negative Metastatic Breast Cancer	NCT01918306	I/II	ER- BC, HER- BC, TNBC, Recurrent BC, Stage IV BC -	Currently recruiting participants	Cisplatin, GDC-0941, a PI3K inhibitor	Not provided
*NeoPHOEBE: PI3k Inhibition in Her2 OverExpressing Breast cancEr: A Phase II, Randomized, Parallel Cohort, Two Stage, Double-blind, Placebo-controlled Study of Neoadjuvant Trastuzumab Versus Trastuzumab + BKM120 in Combination With Weekly Paclitaxel in HER2-positive, PIK3CA Wild-type and PIK3CA Mutant Primary Breast Cancer	NCT01816594	II	HER2+, Newly Diagnosed, Primary BC Neoadjuvant Therapy, Trastuzumab	Currently recruiting participants	BKM120, trastuzumab (trastuzumab = herceptin, a anti-HER2 MoAb), paclitaxel	Not provided
Neoadjuvant Phase II Trial of Paclitaxel in Combination With BKM120 in Endocrine Resistant Clinical Stage II or III Estrogen Receptor-Positive and HER2 Negative Breast Cancer	NCT01953445	II	BC	Not yet open for participant study	Paclitaxel, BKM120	Not provided
A Phase Ib Trial of Gemcitabine and Cisplatin With RAD001 in Patients With Metastatic Triple Negative Breast Cancer Proceeding to an Open Label Randomized Phase II Trial Comparing Gemcitabine/Cisplatin With or Without RAD001.	NCT01939418	I/II	Metastatic BC	Currently recruiting patients	RAD001 (Afinitor everolimus), gemcitabine (a nucleoside analog), cisplatin	Not provided
A Randomized, Double-Blind, Placebo-Controlled Phase II Trial of Weekly Paclitaxel/Bevacizumab +/- Everolimus as First-Line Chemotherapy for Patients With HER2-Negative Metastatic Breast Cancer (MBC)	NCT00915603	II	Metastatic BC	Ongoing, but not recruiting	Everolimus, Bevacizumab (vascular endothelial growth factor A inhibitor), paclitaxel	Not provided
*A Phase II Study Evaluating The Efficacy And Tolerability Of Everolimus (RAD001) In Combination With Trastuzumab And Vinorelbine In The Treatment Of Progressive HER2-Positive Breast Cancer Brain Metastases	NCT01305941	II	HER2+ BC	Recruiting	Everolimus, vinorelbine, trastuzumab (HER2 inhibibitor, MoAb)	Not provided
*Phase II Study of Everolimus in Combination With Exemestane Versus Everolimus Alone Versus Capecitabine in the Treatment of Postmenopausal Women With ER+Locally Advanced, Recurrent, or Metastatic Breast Cancer After Recurrence or Progression on Prior Letrozole or Anastrozole.	NCT01783444	II	BC	Recruiting	Capecitabine (a pro-drug, which is converted to 5-flurouracil (5-FU) which inhibits thymidylate synthase, exemestane, Everolimus	Not provided
*A Phase III Trials Program Exploring the Integration of Bevacizumab, Everolimus (RAD001), and Lapatinib Into Current Neoadjuvant Chemotherapy Regimes for Primary Breast Cancer	NCT00567554	III	BC	Ongoing, but not recruiting	Epirubicin, cyclophosphamide, docetaxel, bevacizumab, paclitaxel, everolimus, trastuzumab, lapatinib	[341,342]
Phase I, Open Label, Dose Escalation Study of the Safety, Tolerability, and Pharmacokinetics of the Combination RAD001 Plus Docetaxel in Patients With Metastatic Breast Cancer	NCT00253318	I	BC	Terminated	Docetaxel, RAD001, dexamethasone, a glucocorticoid /steroidial drugs that has anti-inflammatory, immunosuppressant properties	Not provided
Phase II, Open Label, Dose Escalation Study of the Safety, Tolerability, and Pharmacokinetics of the Combination RAD001 Plus Docetaxel in Patients With Metastatic Breast Cancer	NCT01825265	II	BC	Withdrawn prior to enrollment	Docetaxel, RAD001, Dexamethasone	Not provided
*Phase 1b/2 Single-arm Trial Evaluating the Combination of Lapatinib, Everolimus and Capecitabine for the Treatment of Patients With HER2-positive Metastatic Breast Cancer With CNS Progression After Trastuzumab	NCT01783756	I/II	Central Nervous System Metastases HER2+ BC, Male BC, Recurrent BC, Stage IV BC	Currently recruiting	Lapatinib ditosylate, everolimus, capecitabine	Not provided
*A Phase Ib/II Trial of LEE011 in Combination With Everolimus (RAD001) and Exemestane in the Treatment of Postmenopausal Women With Estrogen Receptor Positive, Her2- Locally Advanced or Metastatic Breast Cancer	NCT01857193	I/II	BC	Currently recruiting	LEE011, a CDK4/6 inhibitor, exemestane, everolimus	Not provided
Phase I/II Study of Weekly Abraxane and RAD001 in Women With Locally Advanced or Metastatic Breast Cancer. A Study of the Cancer Institute of New Jersey Oncology Group (CINJOG)	NCT00934895	I/II	BC	Currently recruiting	Everolimus, abraxane, abraxane is paclitaxel bonded to albumin as a delivery vehicle	Not provided
A Phase Ib/II Study of Cisplatin, Paclitaxel, and RAD001 in Patients With Metastatic Breast Cancer	NCT01031446	I/II	BC	Completed	Cisplatin, everolimus, paclitaxel	Not provided
Everolimus (RAD001) in Combination With Intravenous Carboplatin in Taxane- and Anthracycline-pretreated Patients With Progressive Metastatic Breast Cancer	NCT00930475	I/II	BC	Unknown	RAD001 in combination with carboplatin. Carboplatin is related to cisplatin, but is modified.	Not provided
Open Label Randomized Clinical Trial of Standard Neoadjuvant Chemotherapy (Paclitaxel Followed by FEC) Versus the Combination of Paclitaxel and RAD001 Followed by FEC in Women With Triple Receptor-Negative Breast Cancer (CRAD001C24101)	NCT00499603	II	BC	Completed	Paclitaxel, 5-FU, epirubicin, cyclophosphamide, RAD001	Not provided
A Phase I Study of Cisplatin, Paclitaxel, and RAD001 Patients With Metastatic Breast Cancer	NCT00680758	I	BC	Completed	Cisplatin, everolimus, paclitaxel	Not provided
A Phase II Neo-Adjuvant Study of Cisplatin, Paclitaxel With or Without RAD001 in Patients With Triple-negative Locally Advanced Breast Cancer.	NCT00930930	II	BC	Ongoing, but not recruiting	Cisplatin, everolimus, paclitaxel	Not provided
A Phase I Pilot Study of the Oral mTOR Inhibitor RAD001 in Combination With Capecitabine for Metastatic Breast Cancer	NCT00473005	I	BC	Terminated due to principle investigator leaving sponsor	Capecitabine, RAD001	Not provided
Phase I Study of Combined Temosirolimus, Erlotinib and Cisplatin in Advanced Solid Tumors	NCT00998036	I	TNBC	Completed	Temsirolimus, cisplatin, erlotinib	Not provided
A Phase Ib Study of Combination of Temsirolimus (Torisel®) and Pegylated Liposomal Doxorubicin (PLD, Doxil®/ Caelyx®) in Advanced or Recurrent Breast, Endometrial and Ovarian Cancer	NCT00982631	I	Advanced/Recurrent BC, Endometrial Cancer, Ovarian Cancer	Recruitment is unknown because the information has not been verified recently	Temsirolimus/PLD	Not provided
A Phase I, Open-Label, Multi-center Study to Assess the Safety, Tolerability and Pharmacokinetics of AZD6244 (ARRY-142886) When Given in Combination With Standard Doses of Selected Chemotherapies to Patients With Advanced Solid Tumors	NCT00600496	I	BC, Colon Cancer, Lung Cancer, Melanoma, Kidney Cancer	Ongoing, but not recruiting participants	AZD6244 (MEK inhibitor), dacarbazine, Erlotinib (EGFR1 inhibitor), docetaxel, temsirolimus	[343]
A Phase I Study of Lenalidomide in Combination With Bevacizumab, Sorafenib, Temsirolimus, or 5-fluorouracil, Leucovorin, Oxaliplatin (FOLFOX) in Patients With Advanced Cancers	NCT01183663	I	Advanced Cancers	Ongoing, but not recruiting	Lenalidomide (related to thalidomide), bevacizumab sorafenib (Raf and other kinase inhibitor), temsirolimus, oxaliplatin, leucovorin, (Folinic acid), 5-FU	Not provided
Phase I Study of Pegylated Liposomal Doxorubicin and Temsirolimus in Resistant Solid Malignancies	NCT00703170	I	Resistant Solid Malignancies	Completed	Temsirolimus, pegylated liposomal doxorubicin	Not provided
A Phase I Study of the mTOR Inhibitor Rapamycin (Rapamune, Sirolimus) in Combination With Abraxane (Paclitaxel Protein-bound Particles) in Advanced Solid Cancers	NCT00337376	I	Advanced Solid Cancers	Terminated	Rapamune, Abraxane	Not provided
A Dose-finding Phase Ib Study Followed by an Open-label, Randomized Phase II Study of BEZ235 Plus Paclitaxel in Patients With HER2 Negative, Inoperable Locally Advanced or Metastatic Breast Cancer	NCT01495247	I/II	Inoperable Locally Advanced Breast Cancer, Metastatic MBC	Ongoing, but not recruiting	BEZ235, paclitaxel	Not provided
Phase I Study of Docetaxel and Temsirolimus in Resistant Solid Malignancies	NCT00703625	I	Resistant Solid Malignancies	Completed	Temsirolimus, docetaxel	Not provided

EGFR, PI3K, and mTORC1 inhibitors are being evaluated to treat breast cancer patients. Drug targeting the ER have been developed and many have been evaluated in clinical trials [273]. The ER modulators: 4HT, raloxifene, and lasofoxifene) and AIs (anastrozole, letrozole, and exemestane) have been evaluated extensively [273]. An important pitfall with these drugs is they were thought to be only effective in ER+ cancers. Drugs such as herceptin are more effective on HER2+ breast cancers.

Lapatinib is a small molecule HER2/EGFR1 dual kinase inhibitor that has also shown be effective in inhibiting the growth of HER2+ breast cancers. TNBCs do not express either ER or HER2, thus novel therapies need to be developed for this class of breast cancer. Drugs being evaluated to treat TNBC include poly ADP-ribose polymerase (PARP) inhibitors, vitamin D, and rexinoids, which activate the vitamin D and retinoid X receptors.

### Mechanisms of Lapatinib-Resistance in HER2+ Cells: Activation of PI3K and Src

The PI3K pathway and Src activation may be a mechanism by which some HER2+ cells grow in response to HER2 inhibition by treatment with small molecule HER2 inhibitors such as lapatinib. Lapatinib-resistant HER2+ breast cancer cell lines were generated by culturing the cells in the presence of lapatinib for prolonged periods of time. These cells did not express activated HER2, but they did express the PI3K/PTEN/Akt/mTORC and Raf/MEK/ERK pathways which is believed to be due to activated Src family members. Treatment of the lapatinib-resistant HER2+ cells with Src inhibitors suppressed the PI3K/PTEN/Akt/mTOR pathway as well as growth and restored sensitivity to lapatinib. Treatment of primary HER2+ tumors with lapatinib actually resulted in the expression of Src-family kinases as detected by mRNA analysis. Treatment of HER2+ BT-474 cells with lapatinib and Src inhibitors was more effective in suppressing the growth of these cells in xenograft models than treatment with lapatinib alone. These studies provide a rationale for the treatment of certain HER2+ cells with the combination of lapatinib and Src inhibitors as a means to prevent drug resistance of HER2+ cells [274]. Thus activation of Src family kinases may be a mechanism by which some HER2+ cells become resistant to HER2 inhibitors such as lapatinib.

### Cotargeting of mTORC1 and Other Signaling Pathways in Breast Cancer Therapy

The possibility of targeting mTORC1 and other signaling pathways such as Raf/MEK/ERK, PI3K, IGF-1R to treat breast cancer is also being examined [275]. In some cases, treatment with rapalogs induces the IGF-1R or PDGFR pathways which in turn will activate the Raf/MEK/ERK pathway. This type of approach may also be appropriate to over come the rapamycin-resistance that certain breast cancers have developed. There are clinical trials in progress evaluating the effectiveness of co targeting mTORC1 and pathways such as IGF-1R, PI3K and Raf/MEK/ERK (find out how many trials).

Combinations of Herceptin with pertuzumab, or T-DM1 and mTOR inhibitors added to an aromatase inhibitor are new therapeutic approaches for the treatment of HER2+ breast cancers [276].

Inhibition of mTORC1 in combination with endocrine therapy may be an approach for treatment of metastatic breast cancer patients which are resistant to aromatase in inhibitors. Two clinical trials (TAMRAD and BOLERO-2) have revealed significant effects [277].

### Combining Herceptin with PI3K, mTORC1 Inhibitors or Chemotherapy to Improve Breast Cancer Therapy

Recently it was demonstrated that combining a pan PI3K inhibitor (XL 147) with Herceptin may overcome herceptin-resistance in breast cancer by suppressing HER2/PI3K/FOXO/survivin signaling [278].

Clinical trials have been performed examining the ability to combine lapatinib, herceptin with paclitaxel in first line HER2+ positive breast cancer patients. The dose limiting toxicity observed in the study was diarrhea [279]. Clinical studies examining the combination of the mTORC1 blocker everolimus and either 4HT or exemestane (Aromasin) have been reviewed [280]. Previous clinical studies combining endocrine therapy with rapalogs to treat metastatic hormone receptor+ breast cancer patients yielded variable results. However, two recent independent trials which selected patients refractory to endocrine therapy, demonstrated that combining the rapalog everolimus (Afinitor) with 4HT or combining everolimus with exemestane (Aromasin) was more effective than either endocrine agent alone. The rapalogs may sensitize the normally endocrine therapy resistant patients to endocrine therapy. Likewise additional clinical trials with HER2+ breast cancer patients and the inclusion of PI3K/mTOR inhibitors may improve therapy of the HER2 inhibitors.

### Novel Approaches to Treat TNBC Patients

While therapies for ER+ and HER+ breast cancer patients have improved over the years, the treatment possibilities for TNBC are more limited. A recent review has summarized the clinical results with more novel approaches to treat TNBCs which include inhibitors of enzymes such as PARP and HDAC, kinases such as Jak2 and Src and receptor kinases as well as biological processes such as angiogenesis [281].

### Resistance to Therapy

**Figure 7:**
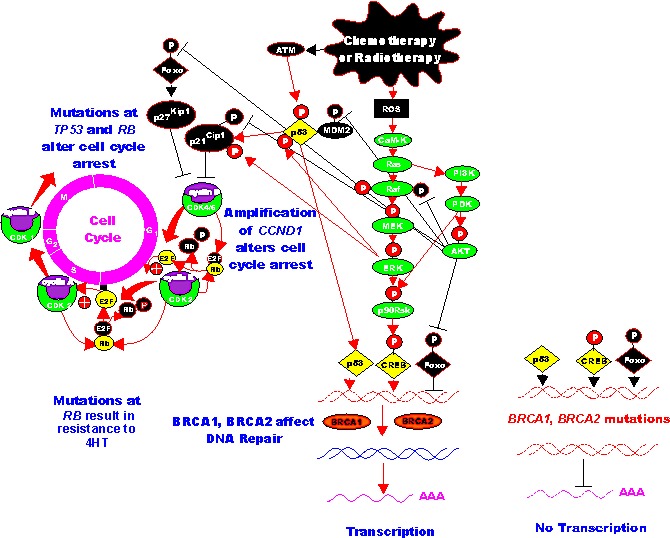
Induction of the Ras/Raf/MEK/ERK Pathway after Leukemia Therapy and Subsequent Effects on Cell Cycle Progression, Survival Pathways and Protein Translation After chemotherapy or radiotherapy there can be activation of signaling pathways which can actually promote cell survival and may lead to therapy resistance. Chemotherapeutic drug treatment (shown in irregular black oval) frequently results in the induction of reactive oxygen species (ROS) (shown in black square). ROS can induce the calcium calmodulin kinase (CaM-K) cascade which can induce Ras which can subsequently activate both the Raf/MEK/ERK and PI3K/PTEN/Akt/mTOR cascades (most components of the two cascades which promote signaling are show in green ovals, transcription factors activated by events are shown in yellow diamonds, transcription factors inactivated by events shown in black diamonds). Induction of the Ras/Raf/MEK/ERK and PI3K/PTEN/Akt/mTOR pathways can result in the activation of many survival pathways, and regulate both cell cycle progression as well as protein translation. Some of the phosphorylation events mediated by Akt actually serve to inhibit the activities of key proteins such as the Foxo transcription factors and the murine double minute (MDM2) ubiquitin ligase (depicted in black ovals). MDM2 serves to regulate p53 protein stability by ubiquitination, however when it is phosphorylated by Akt it is inactivated. Moreover chemotherapeutic drugs and radiotherapy can induce the ataxia telangiectasia mutated (ATM) protein shown in a black oval, which can in turn phosphorylate and regulate p53. p53 can have complex positive and negative effects on cell growth (depicted in yellow diamond), it can regulate the expression of p21 cyclin dependent kinase inhibitory protein-1 (p21^Cip1^) which controls cell cycle progression. p53 can also control the transcription of genes such as Puma, Noxa and Bax which are involved in apoptosis (all of these molecules are shown in black ovals, as they tend to inhibit cell cycle progression or promote apoptosis). Both Akt and ERK can phosphorylate p21^Cip1^ which alters its activity and ability to inhibit cell growth (shown as black phosphorylation sites) and subsequently influence cell growth and therapy resistance. p27^Kip1^ can also be phosphorylated by both Akt and ERK, however the effects of these phosphorylation events are unclear. Akt phosphorylation of p27^Kip1^ may result in its cytoplasmic localization, while ERK phosphorylation of p27^Kip1^ may result in elevated levels of the protein. Hence phosphorylation of proteins by ERK and Akt can have dramatic effects on cell proliferation and contribute to the therapy resistance. Chemo- and radiotherapy also induce breaks in the DNA. In the presence of functional *BRCA1* and *BRCA2* (indicated in yellow ovals) these breaks may be repaired and normal gene transcription can occur. However when *BRCA1* or *BRCA2* are mutated, the repair of these genes may not occur and proper gene transcription might not occur. This figure serves as an introduction as to how activation of these pathways by chemotherapy and radiotherapy may contribute to therapeutic resistance.

Rb is a critical protein involved in the regulation of cell cycle progression and is a tumor suppressor. The Rb pathway is involved in sensitivity to tamoxifen. Loss of functional Rb activity is associated with resistance to tamoxifen [282,284]. Thus oncogenes and tumor suppressor genes often are important in the sensitivity to therapy [168,285]. A diagram depicting some of the sites where mutations result in resistance to therapy is presented in Figure 7. Breast cancers can be inherently drug resistance or develop an acquired resistance after exposure to the particular drug [213,252]. Resistance can develop in patients and breast cancer cells when treated with anthracycline chemotherapy drugs, such as doxorubicin, daunorubicin, and epirubicin as well as hormonal based therapies [199,285,286]. Therefore, cancer cells are capable of undergoing calculated changes that confer survival advantages in otherwise nutrient-restricted or toxic environments [287,288]. Thus novel targets and approaches to treat breast cancer are being evaluated [167,289-308]. TGF-beta and Smads are also important targets in breast cancer [309-312]. The anti-diabetes drug metformin is showing some successes in treating breast cancer including Herceptin-resistant breast cancers [313-317] A target of metformin is AMPK which is involved in regulation of components of the PI3K/Akt/mTORC1 and GSK-3 pathways. An additional target of metformin is Stat3 and it has been shown that metformin can inhibit the growth of TNBC [318]. Additional Stats may be important in the progression of breast cancer [319].

Drug resistance may involve multiple approaches such as pumping the compound out of the cell, modifying or detoxifying the drug, or activating survival signaling pathways that prevent drug-induced apoptosis [287]. ATP-binding cassette transporters such as multidrug resistance protein (MRP1) and the MDR1 product, P-glycoprotein (Pgp), actively expel chemotherapeutic drugs from the cell [239,240]. Drug transporters pumps have been detected in a large number of untreated breast cancers, and their expression increases upon chemotherapy exposure [320]. Certain (e.g., EGFR inhibitors) targeted therapeutics antagonize drug transporter activity [321]. Certain CDK4 inhibitors antagonize the response of breast cancer cells to antracyclines [322]. Activation of cell survival pathways such as PI3K/PTEN/Akt/mTORC1 pathway can prevent apoptosis in the presence of a drug by altering mitochondrial bioenergetics and inhibiting the release of cytochrome c and result in resistance to autophagy [323-329]. The tumor microenvironment and metabolism may influence the response to targeted therapy as well as drug resistance of breast cancer [330]. Genes and biotargets are being identified which can confer resistance or sensitivity to other targeted therapies such as PARP inhibitors [331,332].

### Effects of Metformin on HER2+ Breast CICs which are Resistant to Herceptin

JIMT-1 is a human breast cancer cell line that was isolated from a pleural metastasis of a patient who was resistant to herceptin. CICs were isolated from this cell line JIMT-1(CIC). JIMT-1(CICs) were shown to be preferentially sensitive to the anti-diabetes drug metformin compared to the non-CIC JIMT(BC) population. Furthermore, inclusion of metformin in tumor xenograft studies increased the ability of Herceptin to suppress the growth of JIMT xenografts. These important studies document the potential usefulness of metformin in the treatment of HER2+ breast cancer patients [298].

Similar studies were performed with a herceptin-sensitive (SKBR3-TzbS) and a derivative Herceptin-resistant (SKBR3-TzbR) breast cancer cell lines. While herceptin inhibited the formation of mammospheres from herceptin-sensitive (SKBR3-TzbS), it did not suppress the formation of mammospheres from herceptin-resistant (SKBR3-TzbR) cells. Metformin would reduce the formation of mamospheres from both cell types but the SKBR3-TzbR cells were more sensitive to metformin. Metformin could be combined with herceptin and synergistically reduced mammosphere formation ability in SKBR-TzbR cells [333].

### Induction of EMT can lead to Herceptin Resistance

Induction of EMT in breast cancer is also another mechanism of resistance to such drugs as herceptin [334]. HER2 is also associated with breast cancer stem cells and their aggressiveness [335]. Inherent herceptin resistance in HER2+ breast cancers is a significant problem and it has been reported to be as high as 70% [334]. In the above study, basal HER2+ breast cancer cells resistant to herceptin were infected with lentiviruses containing small hairpin (sh) RNAs specific for EMT-specific transcription factors. The authors had determined appropriate genes to target by analysis of the public Lawrence Berkeley Laboratory (LBL) Breast Cancer Collection database.

These authors demonstrated that herceptin sensitivity was restricted to the SLUG/SNAIL2-negative subset of luminal/HER2+ cell lines. In contrast, breast cancer lines which expressed SLUG/SNAIL2 were inherently resistance to herceptin. Knockdown of SLUG/SNAIL2 suppressed the CIC phenotype by upregulating the expression of the luminal epithelial marker CD24 in basal/HER2+ cells and these cells were also sensitive to herceptin and underwent the mesenchymal to epithelial transition (MET). This important study also documented a reduction in tumor growth and sensitivity to herceptin when SLUG and SNAIL2 were knocked-down in HER+ cells in tumor xenograft studies. This group has also postulated that other mechanisms may be involved in the induction of herceptin-refractory CICs from more differentiated cells via the activation of intrinsic or microenvironmental paths-to-stemness, also involving EMT [336].

### Clinical Trials with Breast Cancer Patients Regarding Key Pathways Discussed

In Tables 1-3 and Supplementary Tables 1-3, we present the listing of clinical trials with breast cancer patients derived from the ClinicalTrials.gov data base. We have focused our searches to the key pathways discussed in this review. Table 1 presents the clinical trials with single inhibitors (mono therapy) such as PI3K, PI3K/mTOR, Akt, mTOR inhibitors and mTORC1 blockers (Everolimus, Temsirolimus, Rapamycin). Some trials have presented publication regarding their clinical trials, either in the form of abstracts or scientific manuscripts [333-340]. In Table 2, we list the trials which have used 2 or more agents, usually a combination of inhibitors which target two different signaling pathways. Table 3 lists clinical trials which often combine a signal transduction inhibitor with chemotherapy. Some of these clinical trails have presented publications in the database [341-343]. In Supplementary Table 1, clinical trials which combine hormonal therapy with targeted or chemotherapy are presented. Some of these clinical trails have presented publications in the database [344-350]. Clinical trials using metformin as a single agent are presented in Supplementary Table 2. Finally clinical trails combining metformin and AIs and other drugs are presented in Supplementary Table 3 as well as a publication from one study [351].

## SUMMARY

HER2, EGFR1, EGFRvIII and the PI3K/PTEN/Akt/mTORC1 pathway are clearly important not only in the development of breast cancer, but also in the classification and prognosis of breast cancer patients which display mutations or changes in the expression of components of these pathways. Both *HER2* and *PIK3CA* are aberrantly expressed or mutated in many breast cancers. These genes may be altered in breast CICs and successful targeting of them may prove to be effectively therapeutically. HER2 and PI3K control the expression of many downstream genes involved in many different biological processes including protein translation and gene transcription.

## Supplementary Tables


