# Supplementary material for: Deregulation of the EGFR/PI3K/PTEN/Akt/mTORC1 pathway in breast cancer: possibilities for therapeutic intervention
Source: Oncotarget. 2014 Jul 12;5(13):4603–50. doi: 10.18632/oncotarget.2209 (PMC4148087; doi:10.18632/oncotarget.2209)
Supplement: Supplementary file 1 [file oncotarget-05-4603-s001.docx]

**Supplementary Table 1: Targeted Agents in combination with Hormonal Therapy**

| **Official Trial Name** | **Clinical Trial #** | **Phase of Trial** | **Type of Cancer Tested** | **Status of Trial** | **Intervention** | **Publications/**  **Results** |
| --- | --- | --- | --- | --- | --- | --- |
| A Phase I Study of MK-2206 in Combination With Lapatinib in Refractory Solid Tumors Followed by Dose-expansion in Advanced HER2+ Breast Cancer | NCT01245205 | I | HER2+ BC, Male BC, Recurrent BC. Stage IIIB BC, Stage IIIC BC, Stage IV BC, Unspecified Adult Solid Tumor | Recruiting | Akt inhibitor MK2206, lapatinib ditosylate | Not  provided |
| A Phase 1b Dose-Escalation Study of the AKT Inhibitor MK-2206 (NSC# 749607) Plus Lapatinib (NSC# 727989) Administered in Patients With HER2 Positive Metastatic Breast Cancer | NCT01281163 | I | ER- BC, ER+ BC, HER2+ BC, PR- BC, PR+ BC, Recurrent BC, Stage IV BC | Recruiting | MK2206, lapatinib ditosylate | Not  provided |
| A Phase II Trial of Neoadjuvant MK-2206 in Combination With Either Anastrozole if Postmenopausal or Anastrozole and Goserelin if Premenopausal in Women With Clinical Stage 2 or 3 PIK3CA Mutant Estrogen Receptor Positive and HER2 Negative Invasive Breast Cancer | NCT01776008 | II | ER+ BC, HER2- BC, Recurrent BC, Stage II BC, Stage IIIA BC, Stage IIIB BC, Stage IIIC BC | Recruiting | Akt inhibitor MK2206, anastrozole (an AI), goserelin acetate (Goserelin acetate suppresses production of the testosterone and estrogen.) | Not  provided |
| A Phase I Study of MK-2206 in Combination With Trastuzumab and Lapatinib in HER2-Positive Breast and Gastric Cancer | NCT01705340 | I | Adenocarcinoma of the Gastroesophageal Junction  HER2+ BC, Male BC, Recurrent BC, Recurrent Esophageal Cancer, Recurrent Gastric Cancer, Stage IIIC BC, Stage IIIC Esophageal Cancer, Stage IIIC Gastric Cancer, Stage IV Breast Cancer, Stage IV Esophageal Cancer, Stage IV Gastric Cancer | Terminated | MK2206, trastuzumab, lapatinib ditosylate | Not  provided |
| A Phase 1 Trial of MK-2206 in Combination With Anastrozole, Fulvestrant, or Anastrozole Plus Fulvestrant in Postmenopausal Women With Estrogen Receptor Positive Metastatic Breast Cancer | NCT01344031 | I | ER+ BC, Recurrent BC, Stage IV BC | Ongoing, but not recruiting | MK2206, anastrozole, fulvestrant | Not  provided |
| Phase II Trial of MK-2206 (an AKT Inhibitor) in Combination With Endocrine Therapy in Patients With Hormone Receptor Positive Breast Cancer | NCT01240941 | II | Metastatic BC | Withdrawn | MK-2206, Exemestane (an AI), goserelin | Not  provided |
| A Phase Ib Trial of MK-2206 (an AKT Inhibitor) in Combination With Endocrine Therapy in Patients With Hormone Receptor-Positive Breast Cancer | NCT01240928 | I | Metastatic BC | Withdrawn prior to enrollment | MK-2206, exemestane, goserelin | Not  provided |
| A Phase Ib Trial of BYL719 (an α-Specific PI3K Inhibitor) in Combination With Endocrine Therapy in Post-Menopausal Patients With Hormone Receptor-Positive Metastatic Breast Cancer | NCT01791478 | I | ER+ BC, HER2- BC, PR+ BC  Invasive Ductal Breast Carcinoma,  Recurrent BC, Stage IV BC | Currently recruiting participants | PI3K inhibitor BYL719, letrozole | Not  provided |
| *NeoPHOEBE: Pi3k Inhibition in Her2 OverExpressing Breast cancEr: A Phase II, Randomized, Parallel Cohort, Two Stage, Double-blind, Placebo-controlled Study of Neoadjuvant Trastuzumab Versus Trastuzumab + BKM120 in Combination With Weekly Paclitaxel in HER2-positive, PIK3CA Wild-type and PIK3CA Mutant Primary Breast Cancer | NCT01816594 | II | HER2+, Newly Diagnosed, Primary BC, Neoadjuvant Therapy, Trastuzumab | Currently recruiting participants | BKM120, trastuzumab, paclitaxel | Not  provided |
| A Phase Ib Trial of BKM120 (a PI3K Inhibitor) or BEZ235 (a PI3K/mTOR Inhibitor) in Combination With Endocrine Therapy in Post-Menopausal Patients With Hormone Receptor-Positive Metastatic Breast Cancer | NCT01248494 | I | Metastatic BC | Completed | BEZ235, BKM 120, Letrozole | Not  provided |
| A Phase II Randomized, Double-blind Placebo Controlled, Study of Letrozole With or Without BYL719 or Buparlisib, for the Neoadjuvant Treatment of Postmenopausal Women With Hormone Receptor-positive HER2-negative Breast Cancer | NCT01923168 | II | BC | Currently recruiting participants | Letrozole, BYL719, BKM120 | Not  provided |
| A Phase Ib/II Open-label Study Evaluating Safety and Efficacy of Oral BKM120 in Combination With Lapatinib in HER2+/PI3K-activated, Trastuzumab-resistant Locally Advanced, Recurrent and Metastatic Breast Cancer. PIKHER2/IPC 2011-001 | NCT01589861 | I/II | BC | Currently recruiting participants | BKM120, lapatinib | Not  provided |
| A Phase II Study of Rapamycin (Rapamune, Sirolimus) and Trastuzumab (Herceptin) for Patients With HER-2 Receptor Positive Metastatic Breast Cancer | NCT00411788 | II | BC | Recruitment is unknown | Rapamycin, trastuzumab | Not  provided |
| A Phase IV Multicentre, Open Label Study of Postmenopausal Women With Oestrogen Receptor Positive Locally Advanced or Metastatic Breast Cancer Treated With Everolimus (RAD001) in Combination With Exemestane, With Exploratory Epigenetic Marker Analysis | NCT01743560 | IV | ER+ Advanced BC | Ongoing, but not recruiting | RAD001, exemestane | Not  provided |
| An Open-label, Phase II, Single-arm Study of Everolimus in Combination With Letrozole in the Treatment of Postmenopausal Women With Estrogen Receptor Positive HER2 Negative Metastatic or Locally Advanced Breast Cancer | NCT01698918 | II | ER+ BC | Currently recruiting participants | Everolimus, letrozole, exemestane | Not  provided |
| A Brief Dose Escalation Followed by a Phase 2 Study of RAD001 in Combination With Trastuzumab in HER2-Positive Metastatic Breast Cancer | NCT00458237 | I/II | BC | Ongoing, but not recruiting | Everolimus, trastuzumab | [345] |
| Phase II Trial of Lapatinib in Combination With Everolimus in Triple Negative Metastatic or Locally Advanced Breast Cancer | NCT01272141 | II | BC | Terminated | Lapatinib, everolimus | Not provided |
| *A Phase II Study Evaluating The Efficacy And Tolerability Of Everolimus (RAD001) In Combination With Trastuzumab And Vinorelbine In The Treatment Of Progressive HER2-Positive Breast Cancer Brain Metastases | NCT01305941 | II | HER2+ BC | Recruiting | Everolimus, vinorelbine, trastuzumab | Not provided |
| A Randomized Phase III, Double-Blind, Placebo-Controlled Multicenter Trial of Everolimus in Combination With Trastuzumab and Paclitaxel, as First Line Therapy in Women With HER2 Positive Locally Advanced or Metastatic Breast Cancer | NCT00876395 | III | BC | Recruiting | Everolimus, trastuzumab | Not provided |
| A Phase I/II Trial of Temsirolimus Plus Neratinib for Patients With Metastatic HER2-Amplified or Triple Negative Breast Cancer | NCT01111825 | I/II | BC | Recruiting | Temsirolimus, Neratinib, a dual EGFR1 and HER2 inhibitor | Not provided |
| Randomized Phase II Study to Compare Vinorelbine In Combination With the mTOR Inhibitor Everolimus vs. Vinorelbin Monotherapy for Second-line Treatment in Advanced Breast Cancer | NCT01520103 | II | HER2- Metastatic BC,  HER2- Locally Advanced BC | Recruiting | Vinorebine, everolimus | Not provided |
| Phase I-II Study of Trastuzumab in Combination With RAD001 in Patients With HER-2 Overexpressing, PTEN-deficient Metastatic Breast Cancer Progressing on Trastuzumab-Based Therapy | NCT00317720 | I/II | BC | Completed | Trastuzumab, RAD001 | Not provided |
| *Phase II Study of Everolimus in Combination With Exemestane Versus Everolimus Alone Versus Capecitabine in the Treatment of Postmenopausal Women With ER+Locally Advanced, Recurrent, or Metastatic Breast Cancer After Recurrence or Progression on Prior Letrozole or Anastrozole. | NCT01783444 | II | BC | Recruiting | Capecitabine, exemestane, everolimus | Not provided |
| A Phase IIIB, Multi-Center, Open Label Study For Postmenopausal Women With Estrogen Receptor Positive Locally Advanced or Metastatic Breast Cancer Treated With Everolimus (RAD001) in Combination With Exemestane: 4EVER - Efficacy, Safety, Health Economics, Translational Research | NCT01626222 | III | Metastatic BC | Ongoing, but not recruiting participants | Exemestane, everolimus | Not  provided |
| A Phase II Study of Combined Fulvestrant (Faslodex) and RAD001 (Everolimus) in Advanced/Metastatic Breast Cancer After Aromatase Inhibitor Failure | NCT00570921 | II | BC | Ongoing, but not recruiting | RAD001, fulvestrant | [345] |
| *A Phase III Trials Program Exploring the Integration of Bevacizumab, Everolimus (RAD001), and Lapatinib Into Current Neoadjuvant Chemotherapy Regimes for Primary Breast Cancer | NCT00567554 | III | BC | Ongoing, but not recruiting | Epirubicin (an anthracycline)- cyclophosphamide / docetaxel, paclitaxel, everolimus, trastuzumab, lapatinib | [341, 342] |
| *Phase 1b/2 Single-arm Trial Evaluating the Combination of Lapatinib, Everolimus and Capecitabine for the Treatment of Patients With HER2-positive Metastatic Breast Cancer With CNS Progression After Trastuzumab | NCT01783756 | I/II | ­Central Nervous System Metastases,  HER2+ BC, Male BC,  Recurrent BC, Stage IV BC | Currently recruiting | lapatinib ditosylate, everolimus, capecitabine | Not  Provided |
| *A Phase Ib/II Trial of LEE011 in Combination With Everolimus (RAD001) and Exemestane in the Treatment of Postmenopausal Women With Estrogen Receptor Positive, Her2- Locally Advanced or Metastatic Breast Cancer | NCT01857193 | I/II | BC | Currently recruiting | LEE011, exemestane, everolimus | Not  Provided |
| Circulating FGF21 Levels and Efficacy of Exemestane, Everolimus and Metformin in Postmenopausal Women With Hormone Receptor Positive Metastatic Breast Cancer and BMI >/= 25 | NCT01627067 | II | BC | Currently recruiting | Everolimus, exemestane metformin | Not  Provided |
| A Randomized Double-Blind, Placebo-Controlled Study of Everolimus in Combination With Exemestane in the Treatment of Postmenopausal Women With Estrogen Receptor Positive Locally Advanced or Metastatic Breast Cancer Who Are Refractory to Letrozole or Anastrozole | NCT00863655 | III | BC | Ongoing, but not recruiting participants | Everolimus, exemestane | Not  Provided |
| A Phase II, Randomized, Multi-center Study, Assessing Value of Adding Everolimus (RAD001) to Trastuzumab as Preoperative Therapy of HER-2 Positive Primary Breast Cancer Amenable to Surgery. | NCT00674414 | II | BC | Terminated due to accrual issue (82 points accrued/120 expected) | Trastuzumab, everolimus | Not  Provided |
| Phase II Open Label Study of Everolimus (RAD001) in Combination With Letrozole in the Treatment of Post Menopausal Women With Locally Advanced or Metastatic Breast Cancer Women With Estrogen Receptor Positive After Failure of Tamoxifen and or Anestrozole or Examestane. | NCT01231659 | IV | Postmenopausal Women, Locally Advanced, or Metastatic Breast Cancer | Ongoing, but not recruiting participants | Everolimus, letrozole | Not  Provided |
| GCC 0901- A Phase II Study of Letrozole in Combination With Lapatinib Followed by an Addition of Everolimus in Postmenopausal Women With Advanced Endocrine Resistant Breast Cancer | NCT01499160 | II | Breast Neoplasms, Endocrine Breast Diseases, Neoplasm Metastasis | Recruiting | Letrozole, lapatinib, everolimus | Not  Provided |
| Randomized, Double-Blind, Placebo-Controlled Phase II Trial of Fulvestrant (Faslodex) Plus Everolimus in Post-Menopausal Patients With Hormone-Receptor Positive Metastatic Breast Cancer Resistant to Aromatase Inhibitor Therapy | NCT01797120 | II | Metastatic BC | Currently recruiting | Fulvestrant, everolimus | Not  provided |
| Randomized Phase II Trial of Trastuzumab or EVEROLIMUS in Hormone-refractory Metastatic Breast Cancer | NCT00912340 | II | BC | Currently recruiting | Trastuzumab (Herceptin), everolimus | Not  Provided |
| A Randomized Phase III, Double-blind, Placebo-controlled Multicenter Trial of Daily Everolimus in Combination With Trastuzumab and Vinorelbine, in Pretreated Women With HER2/Neu Over-expressing Locally Advanced or Metastatic Breast Cancer. | NCT01007942 | III | HER2/Neu Over-expressing Locally Advanced BC, Metastatic BC | Ongoing, but not recruiting | Everolimus, vinorelbine, trastuzumab | Not  Provided |
| Phase III Randomized, Placebo-Controlled Clinical Trial Evaluating the Use of Adjuvant Endocrine Therapy +/- One Year of Everolimus in Patients With High-Risk, Hormone Receptor-Positive and HER2/Neu Negative Breast Cancer | NCT01674140 | III | BC | Recruiting | Anastrozole, everolimus, exemestane, goserelin acetate, letrozole, leuprolide acetate, tamoxifen citrate | Not  provided |
| A Phase 2, Double-blind, Randomized, Placebo-controlled, Multi-center Study Assessing the Value of Adding Everolimus to Letrozole as Preoperative Therapy of Primary Breast Cancer in Postmenopausal Women | NCT00107016 | II | BC | Completed | RAD001, letrozole | [337]  [.](http://www.clinicaltrials.gov/ct2/bye/xQoPWw4lZX-i-iSxuBcyeXNxvdDxuQ7Ju6c9cX-3LBNLz6YqaKCROR4VWKC95d-3Ws8Gpw-PSB7gW.) |
| A Phase 2 Randomized Open-Label Study Of Letrozole In Combination With Two Dose Levels And Schedules Of Oral Temsirolimus (CCI-779), Or Letrozole Alone, In Postmenopausal Women With Locally Advanced Or Metastatic Breast Cancer | NCT00062751 | II | BC | Completed | Letrozole, temsirolimus (CCI-779) | Not provided |
| Phase II Trial of Lapatinib and RAD-001 for HER2 Positive Metastatic Breast Cancer | NCT01283789 | II | Metastatic BC | Recruiting | Lapatinib, RAD-001 | Not provided |
| A Phase 2 Randomized Open-Label Study Of Letrozole In Combination With Two Dose Levels And Schedules Of Oral Temsirolimus (CCI-779), Or Letrozole Alone, In Postmenopausal Women With Locally Advanced Or Metastatic Breast Cancer | NCT00061971 | II | BC | Completed | Letrozole, temsirolimus (CCI-779) | Not provided |
| A Phase 3 Randomized, Placebo-Controlled, Double-Blind Study of Oral CCI-779 Administered in Combination With Letrozole vs. Letrozole Alone as First Line Hormonal Therapy in Postmenopausal Women With Locally Advanced or Metastatic Breast Cancer | NCT00083993 | III | Breast Neoplasms, Neoplasm Metastasis | Terminated | Temsirolimus (CCI-779) for 34 months, letrozole for 34 months | [346] |
| Phase I Clinical Trial of Temsirolimus and Vinorelbine in Advanced Solid Tumors. | NCT01155258 | I | Extensive Stage Small Cell Lung Cancer, -Hereditary Paraganglioma, Male BC, Malignant Paraganglioma, Metastatic Gastrointestinal Carcinoid Tumor, Metastatic Pheochromocytoma, Pancreatic Polypeptide Tumor, Recurrent BC, Recurrent Cervical Cancer, Recurrent Endometrial Carcinoma, Recurrent Gastrointestinal Carcinoid Tumor, Recurrent Islet Cell Carcinoma, Recurrent Neuroendocrine, Carcinoma of the Skin Recurrent Non-small Cell Lung Cancer, Recurrent Ovarian Epithelial Cancer,  Recurrent Ovarian Germ Cell Tumor, Recurrent Pheochromocytoma, Recurrent Prostate Cancer, Recurrent Renal Cell Cancer, Recurrent Small Cell Lung Cancer, Recurrent Uterine Sarcoma. Regional Gastrointestinal Carcinoid Tumor, Regional Pheochromocytoma, Stage III Cervical Cancer, Stage III Endometrial Carcinoma, Stage III Neuroendocrine Carcinoma of the Skin, Stage III Ovarian Epithelial Cancer, Stage III Ovarian Germ Cell Tumor, Stage III Prostate Cancer, Stage III Renal Cell Cancer, Stage III Uterine Sarcoma, Stage IIIA Breast Cancer, Stage IIIA Non-small Cell Lung Cancer, Stage IIIB Breast Cancer, Stage IIIB Non-small Cell Lung Cancer, Stage IIIC BC, Stage IV BC, Stage IV Endometrial Carcinoma, Stage IV Neuroendocrine Carcinoma of the Skin, Stage IV Non-small Cell Lung Cancer, Stage IV Ovarian Epithelial Cancer, Stage IV Ovarian Germ Cell Tumor, Stage IV Prostate Cancer, Stage IV Renal Cell Cancer, Stage IV Uterine Sarcoma, Stage IVA Cervical Cancer, Stage IVB Cervical Cancer, Thyroid Gland Medullary Carcinoma | Ongoing, but not recruiting | Temsirolimus, vinorelbine ditartrate | Not  provided |
| A Phase III Randomized, Double Blind, Placebo Controlled Study of BKM120 With Fulvestrant, in Postmenopausal Women With Hormone Receptor-positive HER2-negative AI Treated, Locally Advanced or Metastatic Breast Cancer Who Progressed on or After mTOR Inhibitor Based Treatment | NCT01633060 | III | Metastatic BC, HR+, HER2- | Recruiting | Fulvestrant, BKM120 | Not  provided |
| Phase I Study of Combined Temosirolimus, Erlotinib and Cisplatin in Advanced Solid Tumors | NCT00998036 | I | TNBC | Completed | Temsirolimus, cisplatin, erlotinib | Not  provided |
| An Open-Label Randomized Phase 2 Study Of PF-04691502 (PI3K/mTOR Inhibitor) In Combination With Exemestane Compared With Exemestane Alone In Patients With Estrogen Receptor Positive, Her-2 Negative Advanced Breast Cancer | NCT01658176 | II | BC | Withdrawn | PF-04691502, Exemestane | Not  provided |
| A Phase I, Open-Label, Multi-center Study to Assess the Safety, Tolerability and Pharmacokinetics of AZD6244 (ARRY-142886) When Given in Combination With Standard Doses of Selected Chemotherapies to Patients With Advanced Solid Tumors | NCT00600496 | I | BC, Colon Cancer, Lung Cancer, Melanoma, Kidney Cancer | Ongoing, but not recruiting participants | AZD6244, dacarbazine, erlotinib, docetaxel, temsirolimus | [347] |
| A Phase Ib Trial of BKM120 (a PI3K Inhibitor) or BEZ235 (a PI3K/mTOR Inhibitor) in Combination With Endocrine Therapy in Post-Menopausal Patients With Hormone Receptor-Positive Metastatic Breast Cancer | NCT01248494 | I | Metastatic BC | Completed | BEZ235, BKM 120, letrozole | Not  provided |
| Phase 2 Study Assessing the Tolerance and Efficacy of Tamoxifen Alone Versus the Association Tamoxifen-RAD001 (Everolimus) in Patients With Anti-aromatase Resistant Breast Metastatic Cancer | NCT01298713 | II | BC | Recruitment is unknown because information has not been verified recently | Tamoxifen, everolimus | Not  provided |
| A Phase II Trial of Oral Deforolimus (AP23573; MK-8669), an mTOR Inhibitor, in Combination With Trastuzumab for Patients With HER2-positive Trastuzumab-Refractory Metastatic Breast Cancer | NCT00736970 | II | BC | Completed | Ridaforolimus, trastuzumab | Not  provided |
| A Two-Part Adaptive, Randomized Trial of Ridaforolimus in Combination With Dalotuzumab Compared to Exemestane or Compared to Ridaforolimus or Dalotuzumab Monotherapy in Estrogen Receptor Positive Breast Cancer Patients | NCT01234857 | II | BC | Completed | Ridaforolimus, dalotuzumab, exemestane | Not  provided |
| A Phase II Randomized Trial of the Combination of Ridaforolimus and Exemestane, Compared to Ridaforolimus, Dalotuzumab and Exemestane in High Proliferation, Estrogen Receptor Positive Breast Cancer Patients | NCT01605396 | II | BC | Ongoing, but not recruiting | Ridaforolimus, dalotuzumab, exemestane | Not  provided |

* Fit in more than one category

**Supplementary** **Table 2: Metformin Monotherapy**

| **Official Trial Name** | **Clinical Trial #** | **Phase of Trial** | **Type of Breast Cancer Patient** | **Status of Trial** | **Intervention** | **Publications/Results** |
| --- | --- | --- | --- | --- | --- | --- |
| Phase II Study of Metformin for Reduction of Obesity-Associated Breast Cancer Risk | NCT02028221 | II | BC Prevention | Currently recruiting participants | Metformin | Not  provided |
| The Use of Metformin in Early Breast Cancer Patients Pre-Surgery: A Phase 0 Study Regarding The Biological Effect | NCT01302002 | 0 | BC | Recruitment unknown because the information has not been verified recently | Metformin | Not  provided |
| A Phase 2 Single Arm Study to Examine the Effects of Metformin on Cancer Metabolism in Patients With Early Stage Breast Cancer Receiving Neoadjuvant Chemotherapy | NCT01266486 | II | BC | Currently recruiting participants | Metformin | Not  provided |
| Clinical and Biologic Effects of Metformin in Early Stage Breast Cancer | NCT00897884 | Not provided | BC | Completed | Metformin | Not  provided |
| Pre-Surgical Trial of Metformin in Patients With Operable Breast Cancer | NCT00984490 | Not provided | BC | Withdrawn due to slow accrual | Metformin | Not  provided |
| A Phase III Randomized Trial of Metformin Versus Placebo on Recurrence and Survival in Early Stage Breast Cancer | NCT01101438 | III | BC | Ongoing but not recruiting | Drug: Metformin Hydrochloride Drug: Placebo | Not  provided |
| A Double Blind Prospective Study of Metformin vs. Placebo in Overweight or Obese Post-menopausal Women at Elevated Risk for Breast Cancer | NCT01793948 | Not provided | BC, Obesity | Currently recruiting participants | Metformin | Not  provided |
| Change in Mammographic Density With Metformin Use: A Companion Study to NCIC Study MA.32 | NCT01666171 | Observational | BC | Not yet open for participant recruitment | Metformin | Not  provided |
| Randomized Phase II Study of Exercise and Metformin in Colorectal and Breast Cancer Survivors | NCT01340300 | II | Colorectal Cancer, BC | Currently recruiting participants | Metformin | Not  provided |
| Biobehavioral Mechanisms of Fatigue in Patients Treated on NCIC CTG MA.32: A Phase III Randomized Trial of Metformin Versus Placebo on Recurrence and Survival in Early Stage Breast Cancer | NCT01286233 | Observational | BC | Ongoing, but not recruiting participants | Metformin | Not  provided |
| Testing for Atypia in Random Periareolar Fine Needle Aspiration (RPFNA) Cytology After 12 Months Metformin (1,1-Dimethylbiguanide Hydrochloride) Chemoprevention Versus Placebo Control in Premenopausal Women | NCT01905046 | III | Atypical Ductal Breast Hyperplasia, BRCA1 Mutation Carrier, BRCA2 Mutation Carrier, Ductal Breast Carcinoma in situ,Lobular Breast Carcinoma in situ | Not yet open for participant recruitment | Metformin | Not  provided |
| Obesity-related Mechanisms and Mortality in Breast Cancer Survivors | NCT01302379 | Not provided | BC | Currently recruiting participants | Drug: Metformin  Drug: Placebo | Not  provided |
| Efficacy and Safety of Adjuvant Metformin for Operable Breast Cancer Patients | NCT00909506 | II | BC | Recruitment is unknown because the information has not been verified recently | Metformin | Not  provided |
| Phase II Pre-Surgical Intervention Study for Evaluating the Effect of Metformin on Breast Cancer Proliferation | NCT00930579 | II | BC | Ongoing, but not recruiting participants | Metformin | Not  provided |
| A Pilot Study of Metformin in Patients With a Diagnosis of Li-Fraumeni Syndrome | NCT01981525 | I | -Li-Fraumeni Syndrome | Currently recruiting participants | Metformin | Not  provided |
| A Randomized, Placebo-controlled, Double-blind Multicenter Phase II Study to Investigate the Protectivity and Efficacy of Metformin Against Steatosis in Combination With FOLFIRI and Cetuximab in Subjects With First-line Palliative Treated, KRAS-Wild-Type, Metastatic Colorectal Cancer | NCT01523639 | II | Colorectal Cancer, Steatohepatitis | Terminated due to slow recruitment | Metformin | Not  provided |

**Supplementary Table 3: Metformin Combination Therapy**

| **Official Trial Name** | **Clinical Trial #** | **Phase of Trial** | **Type of Cancer Tested** | **Status of Trial** | **Intervention** | **Publications/Results** |
| --- | --- | --- | --- | --- | --- | --- |
| Pre-Surgical "Window of Opportunity" Trial of the Combination of Metformin and Atorvastatin in Newly Diagnosed Operable Breast Cancer | NCT01980823 | 0 | BC | Currently recruiting | Metformin, atorvastatin (Lipitor, a statin) | Not  provided |
| I-SPY 2 Trial (Investigation of Serial Studies to Predict Your Therapeutic Response With Imaging And moLecular Analysis 2) | NCT01042379 | II | BC | Currently recruiting participants | Paclitaxel,doxorubicin, cyclophosphamide, AMG 386 (An angiopoietin (Ang) 1 and 2 neutralizing peptibody), AMG 479 (Ganitumab, an anti-IGFR1 MoAb), metformin, MK-2206, Trastuzumab, T-DM1 (ado-trastuzumab emtansine), pertuzumab (anti-HER2 MoAb). | Not  provided |
| A Phase I Study of Temsirolimus in Combination With Metformin in Advanced Solid Tumours | NCT00659568 | I | BC, Endometrial Cancer, Kidney Cancer, Lung Cancer, Lymphoma,-Unspecified Adult Solid Tumor | Completed | Metformin, temsirolimus | Not  provided |
| Neoadjuvant Treatment of TEC Versus TEC Plus Metformin in Breast Cancer：A Prospective, Randomized Trial | NCT01929811 | II | BC | Currently recruiting participants | Metformin, docetaxel, epirubicin, cclophosphomide | Not  provided |
| A Randomized Phase II, Double Blind, Trial of Standard Chemotherapy With Metformin (vs Placebo) in Women With Metastatic Breast Cancer Receiving First or Second Line Chemotherapy With Anthracycline, Taxane, Platinum or Capecitabine Based Regimens | NCT01310231 | II | Metastatic BC | Currently recruiting participants | Metformin, anthracyline, taxane, platinum, capecitabine | Not  provided |
| Phase II Randomized Study of Neoadjuvant Metformin Plus Letrozole vs Placebo Plus Letrozole for ER-positive Postmenopausal Breast Cancer | NCT01589367 | II | ER+ BC | Currently Recruiting participants | Metformin, etrozole | [348] |
| Circulating FGF21 Levels and Efficacy of Exemestane, Everolimus and Metformin in Postmenopausal Women With Hormone Receptor Positive Metastatic Breast Cancer and BMI >/= 25 | NCT01627067 | II | BC | Ongoing, but not recruiting participants | Everloimus, exemestane, metoformin | Not  provided |
| Phase I Study of Erlotinib and Metformin in Triple Negative Breast Cancer | NCT01650506 | I | BC | Currently recruiting participants | Metformin, Erlotinib | Not  provided |
| Clinical Phase II, Randomized, Double Blind Trial, to Evaluate the Efficacy of Metformin and Chemotherapy Versus Placebo Nad Chemotherapy in Neoadjuvant Setting for Locally Advanced Breast Cancer | NCT01566799 | II | Locally Advanced BC | Recruitment unknown because the information has not been verified recently | Metformin, paclitaxel  FAC (cyclophosphamide, doxorubicin, 5-FU) | Not  provided |
| Myme: Phase Ii Comparative Study Of Myocet Plus Cyclophosphamide Plus Metformin Versus Myocet Plus Cyclophosphamide In First Line Treatment Of Her2 Negative Metastatic Breast Cancer Patients | NCT01885013 | II | -HER2- metastatic BC | Currently recruiting participant | Metformin, Myocet (a non-pegylated liposomal doxorubicin), cyclophosphamide | Not  provided |
| The Impact of Obesity and Obesity Treatments on Breast Cancer: A Phase I Trial of Exemestane With Metformin and Rosiglitazone for Postmenopausal Obese Women With ER+ Metastatic Breast Cancer | NCT00933309 | I | BC | Completed | Exemestane,  Avandamet (Metformin + Rosiglitazone)  Rosiglitazone is an [insulin](http://en.wikipedia.org/wiki/Insulin) sensitizer, It binds the [PPAR](http://en.wikipedia.org/wiki/PPAR) receptors in fat cells and makes more sensitive to insulin | Not  provided |
| Modulation of Response to Hormonal Therapy With Lapatinib and/or Metformin in Patients With HER2-negative, ER and/or PgR Positive Metastatic Brest Cancer With Progressive Disease After First-line Therapy | NCT01477060 | II | Metastatic BC | Terminated due to insufficient accrual | Lapatinib,  metformin | Not  provided |
